# Metabolic and Synthetic Dysfunction in Acute Fatty Liver of Pregnancy: Reappraising the Swansea Criteria—A Narrative Review

**DOI:** 10.3390/metabo16100799

**Published:** 2026-10-21

**Authors:** Iustina Petra Solomon Condriuc, Razvan Popovici, Eric Oliviu Cosovanu, Raluca Anamaria Mogos, Raluca Haba, Vasile Adrian Sescu, Raluca Stefana Stan, Simona Juliette Mogos, Catalin Mihai Buzduga, Alexandru Carauleanu

**Affiliations:** “Grigore T. Popa” University of Medicine and Pharmacy, 700115 Iasi, Romania; condriuc.iustina-petra@d.umfiasi.ro (I.P.S.C.); razvan.popovici@umfiasi.ro (R.P.); raluca-anamaria-i-mogos@d.umfiasi.ro (R.A.M.); raluca.haba@umfiasi.ro (R.H.); vasile-adrian.sescu@d.umfiasi.ro (V.A.S.); stefana-raluca.stan@d.umfiasi.ro (R.S.S.); simona.mogos@umfiasi.ro (S.J.M.); catalin.buzduga@umfiasi.ro (C.M.B.); ale.carauleanu@umfiasi.ro (A.C.)

**Keywords:** acute fatty liver of pregnancy, Swansea criteria, fatty acid oxidation, long-chain 3-hydroxyacyl-CoA dehydrogenase, mitochondrial dysfunction, metabolic biomarkers, acylcarnitines, HELLP syndrome, early diagnosis, hepatic synthetic function

## Abstract

Acute fatty liver of pregnancy (AFLP) is a rare microvesicular steatotic liver disease of late gestation, linked to impaired mitochondrial fatty acid β-oxidation, in which management centres on recognition and delivery before overt hepatic and multiorgan failure has developed. The Swansea criteria, which require six or more of fourteen features in the absence of an alternative explanation, define AFLP in guidelines and in most published cohorts. This narrative review examines how the criteria behave across the course of the illness and which markers might signal the disease earlier. The criteria are frequently fulfilled once AFLP is clinically established, but their accuracy against an independent reference standard derives from one small, high-prevalence biopsy series, and their specificity against haemolysis, elevated liver enzymes and low platelets (HELLP) syndrome and other causes of acute liver failure appears limited. Because several criteria index established organ failure, the threshold may remain unmet earlier in the illness, a hypothesis consistent with limited retrospective evidence; to our knowledge, no study has evaluated the criteria prospectively at initial presentation. We relate each criterion to hepatic energy failure and appraise candidate early markers, including coagulation indices, antithrombin, conjugated hyperbilirubinaemia, hypocholesterolaemia, soluble fms-like tyrosine kinase-1 and metabolite-level signals of fatty acid oxidation failure; none has a validated early diagnostic threshold. We outline a conceptual, hypothesis-generating framework for prospective research that would complement, not replace, the Swansea case definition.

## 1. Introduction

Acute fatty liver of pregnancy (AFLP) is uncommon, and it is dangerous in a specific way: the interval between a woman feeling unwell and her developing coagulopathy, hypoglycaemia, renal failure and encephalopathy can be measured in days. National surveillance in the United Kingdom identified 57 cases among an estimated 1,132,964 maternities, an incidence of 5.0 per 100,000 (95% confidence interval [CI] 3.8–6.5). Sixty per cent of affected women were admitted to intensive care and 15% to a specialist liver unit [1]. Other estimates range from roughly 1 in 7000 to 1 in 20,000 pregnancies, varying with population and access to obstetric care [2]. The single prospective study of liver dysfunction in pregnancy that gave rise to the current diagnostic criteria found an incidence of about 1 in 875 deliveries (5 in 4377) in southwest Wales [3]. The disparity is itself informative. It reflects ascertainment; referral pattern; and, above all, the threshold at which a clinician is willing to call the illness AFLP.

Maternal survival has improved substantially. Contemporary cohorts report case fatality of 1.8% (95% CI 0–9.4) in the United Kingdom [1], 1.1% in a recent Chinese series of 92 women [4] and 3.8% among 133 women in another [5]. Tertiary referral cohorts enriched for liver failure still report figures near 17% [6]. Perinatal loss remains high: perinatal mortality was 104 per 1000 births in the United Kingdom Obstetric Surveillance System (UKOSS) cohort [1], and a long-running single-centre series reported 120 stillbirths per 1000 pregnancies [7]. The improvement is generally attributed to earlier recognition and prompt delivery rather than to any specific therapy, since no treatment other than delivery and organ support has been shown to alter the natural history [8,9,10].

That attribution places a heavy burden on the diagnostic process. The instrument that carries it is the Swansea criteria: six or more of fourteen clinical, laboratory, radiological and histological features, in the absence of another explanation [3]. These criteria now define AFLP in guidelines [11,12], in surveillance [1], in almost every published cohort [6,13,14] and, increasingly, at the bedside. They are reproduced, with minor variation, in guidance from European, North American, Chinese and international bodies [12,15,16,17]. They have been remarkably useful, but they are now asked to do work for which they were not constructed.

The criteria were derived to classify a small number of prospectively identified cases within one obstetric population. They were not derived to separate early AFLP from its mimics, to be applied at first presentation or to grade severity—yet each of these uses is now encountered in practice or research. The distinction matters because several of the fourteen items are not features of the initiating hepatic lesion at all. Encephalopathy, ascites and a creatinine above 150 µmol/L are consequences of established liver and multiorgan failure, and liver histology, in most published practice, is obtained only postpartum or post-mortem, if at all. A criteria set weighted towards such items can be expected to identify AFLP most reliably once the illness has become clinically unmistakable.

This review therefore asks a narrower and more clinically consequential question than the general reviews of AFLP that already exist: how might the Swansea criteria behave before the illness is fully expressed? We integrate four studies that are usually cited separately. The criteria-validation literature is largely hepatological; the coagulation literature comes mainly from Japanese and Australasian obstetric medicine. To these, we add the metabolic and mechanistic work on foetal and placental fatty acid oxidation and hepatic lipotoxicity, which frames AFLP as a disorder of hepatic energy metabolism, and the small body of work on diagnostic models built from variables available on admission. Our objective is threefold: to appraise the operating characteristics of the Swansea criteria as a function of disease stage; to identify which candidate markers, including metabolic readouts of hepatic energy failure, plausibly precede criteria fulfilment, and how strong the evidence for each actually is; and to specify design principles and validation requirements for stage-specific early-disease criteria.

### Approach to Literature Identification and Certainty Assessment

This review followed a narrative, concept-driven approach in which the literature was identified iteratively rather than through a protocol-registered systematic search. PubMed/MEDLINE was the bibliographic database searched; Europe PMC served as an additional interface for searching these records and for accessing full texts, and Crossref and publisher records served as supplementary resources for locating sources and verifying bibliographic details, not as databases equivalent to MEDLINE. Embase, Scopus and the Cochrane Library were not searched separately. Terms were combined across five conceptual domains: the disease (“acute fatty liver of pregnancy”, “obstetric acute liver failure” and “microvesicular steatosis”), which defined the scope common to all objectives of the review; the diagnostic construct (“Swansea criteria”, “diagnostic criteria” and “diagnostic accuracy”), relevant to diagnostic validation and timing; the principal differential diagnoses (“HELLP syndrome”, “pre-eclampsia”, “hepatitis E”, “thrombotic microangiopathy” and “haemophagocytic lymphohistiocytosis”), relevant to differential diagnosis and specificity; the mechanism (“long-chain 3-hydroxyacyl-CoA dehydrogenase”, “trifunctional protein”, “fatty acid oxidation”, “mitochondrial dysfunction”, “acylcarnitine” and “metabolomics”), relevant to metabolic mechanisms; and candidate markers (“antithrombin”, “fibrinogen”, “hypoglycaemia”, “soluble fms-like tyrosine kinase-1” and “elastography”), relevant to early recognition. Backward and forward citation tracking from landmark papers, guidelines and relevant reviews was pursued until the principal controversies could be represented fairly. The date of the last search, the complete search strings, any limits applied and record counts are not reported.

Source selection was guided by relevance to the review’s stated questions. Reports addressing diagnostic validation, the timing of biochemical abnormalities, metabolic mechanisms, candidate biomarkers or clinically relevant differential diagnoses were considered, with priority given to primary studies for quantitative findings. Guidelines were cited for current recommendations and case definitions, mechanistic and experimental studies for pathophysiology, reviews for context and for pooled data where they were the most complete source, and case reports for observations not otherwise documented. Emphasis was placed on contemporary evidence, while older studies were retained where they established a concept or remain the original source of a finding, and every reference was checked against its original bibliographic record. The sources cited, all published in English, date from 1983 to 2026. Literature identification and selection were iterative and concept-driven, aiming to represent the principal evidence and controversies relevant to the review’s questions rather than to enumerate an exhaustively screened set of records. No formal screening, risk-of-bias instrument or quantitative synthesis was applied. Interpretation considered patient selection, the timing of measurements, the independence of the reference standard, the choice of comparators, potential overlap between predictors and outcomes, and the availability of independent validation. Diagnostic accuracy against a reference standard, criterion fulfilment in cohorts defined by the same criteria and prognostic prediction within already diagnosed populations were treated as distinct forms of evidence. The characteristics and principal findings of the cohorts and series that inform the quantitative statements below are tabulated in Supplementary Table S1. Reporting was guided by the Scale for the Assessment of Narrative Review Articles (SANRA) [18]; Supplementary Table S5 lists each SANRA item and where it is addressed in the manuscript.

The certainty of the statements summarised further was classified into four qualitative categories. Established denotes a well-supported finding with reasonably consistent independent evidence, or a mathematical relationship; the designation applies to the specific statement, not automatically to early diagnostic usefulness. Probable denotes credible clinical evidence with important limitations in design, precision, replication or applicability. Emerging denotes preliminary clinical findings requiring independent confirmation. Knowledge gap denotes insufficient direct evidence to answer the stated question reliably. Assignments considered study design, sample size and precision, the reference standard, the comparators, replication, consistency, the timing of measurement relative to diagnosis and major sources of bias, and a replicated association was distinguished from a validated diagnostic threshold. These categories are qualitative judgements by the authors, not formal Grading of Recommendations Assessment, Development and Evaluation (GRADE) ratings. The qualitative evidence appraisal involved several authors contributing to methodology, formal analysis, validation and critical review. Differences in interpretation were discussed collectively and resolved through informal consensus. No independent duplicate rating or formal inter-rater agreement assessment was undertaken.

## 2. The Swansea Criteria as an Artefact of Their Derivation

Understanding what the criteria can and cannot do requires attention to how they came about. Ch’ng and colleagues studied liver dysfunction prospectively across 4377 deliveries in southwest Wales over 15 months. Abnormal liver tests occurred in 142 women, approximately 3% of deliveries. Pre-eclampsia was present in 68 of them but was the only identifiable cause of liver dysfunction in 15; the syndrome of haemolysis, elevated liver enzymes and low platelets (HELLP syndrome; 30 women, 29 of whom had pre-eclampsia) was the commonest diagnosis, obstetric cholestasis accounted for 23 and sepsis for 17, and AFLP was diagnosed in five [3]. The list of fourteen features and the threshold of six were an operational device for classifying those cases consistently, in an era when liver biopsy was falling out of use. Across the whole cohort of women with liver dysfunction, there were no maternal deaths and only two intrauterine deaths, which is worth remembering: the criteria were framed around a spectrum of severity that included disease of modest severity.

Three structural properties follow from that origin.

First, the fourteen items are heterogeneous in kind. They mix symptoms (vomiting, abdominal pain and thirst) with a neurological sign, eight laboratory thresholds, an imaging finding and a histological finding [3,11,19]. Items of such different provenance are not exchangeable, yet each contributes a single unweighted point. A woman who has vomiting, abdominal pain, thirst, a mildly raised bilirubin, a leucocytosis and a transaminase of 45 IU/L reaches the threshold without any evidence of hepatic failure. A woman with an international normalised ratio (INR) of 2.0, a fibrinogen of 1.2 g/L and a bilirubin of 90 µmol/L may not.

Second, the thresholds were set against general adult reference intervals rather than pregnancy-specific ones. Morton and Laurie examined this directly. Five of eleven case series reporting laboratory values in AFLP had used values divergent from those specified in the criteria. They recommended pregnancy-specific and laboratory-specific reference intervals and suggested that simpler criteria based on hepatocellular damage and hepatic synthetic dysfunction may be an alternative that merits further study [20]. The direction of the problem differs by analyte. A leucocyte count above 11 × 10^9^/L is common in normal labour, so that item adds little. Urate rises through normal gestation and is a recognised feature of pre-eclampsia, so it pulls towards a competing diagnosis. A creatinine above 150 µmol/L is a marked elevation against the low creatinine of normal pregnancy; that item probably carries more discriminatory information than most, but it typically appears late.

Third, and most consequential for validation, the criteria contain their own reference standard. Microvesicular steatosis on liver biopsy is item fourteen. Any criterion count that includes this item and is then validated against biopsy is partly circular because one component of the index test is the reference standard itself. The only biopsy-anchored validation we identified avoided this by design: its investigators describe antenatal application of the remaining thirteen items, with histology obtained afterwards, postnatally or post-mortem [21,22]. The circularity is therefore a property of the construct rather than a demonstrated bias in that study, but it applies wherever histology is counted when available, and it obscures how rarely histology now contributes. No liver biopsies were performed in a series of 133 women with AFLP [5], and no biopsy data are reported in a two-centre cohort of 290, although histology is listed among the criteria applied [6]. Where biopsy is obtained, it is generally performed postpartum, as in a recent case biopsied on the eighteenth postoperative day [23]. Guidelines now state that it is rarely required [12], although reviews of liver biopsy in pregnancy note that histology may still be needed when non-invasive assessment is inconclusive [24].

## 3. What the Validation Evidence Does and Does Not Show

### 3.1. The Single Biopsy-Anchored Validation

The performance figures quoted in guidelines and reviews rest almost entirely on one small study. Goel and colleagues assessed the criteria in 34 women with suspected pregnancy-related liver disease who underwent liver biopsy at a single centre in Vellore, India, between 1998 and 2006, of whom 24 were analysed after ten exclusions. For the prediction of hepatic microvesicular steatosis, they reported a sensitivity of 100%, a specificity of 57%, a positive predictive value (PPV) of 85% and a negative predictive value (NPV) of 100% [22]. The American College of Gastroenterology (ACG) guideline reproduced those predictive values and reported the investigators’ view that the criteria could obviate the need for liver biopsy [11]. A subsequent account of the same study, written by the same investigators with the originator of the criteria, describes the analysed sample as 24 patients biopsied either in the immediate postnatal period (*n* = 19) or post-mortem (*n* = 5), and states that antenatal application of the remaining thirteen items predicts microvesicular steatosis on biopsy [21].

The original letter also gives the underlying counts: 17 of the 24 analysed patients had microvesicular steatosis on biopsy, all of whom met the criteria, as did 3 of the 7 without steatosis [22]. These counts correspond to a prevalence of approximately 71% in the validation sample. Predictive values are functions of prevalence and cannot be directly transported to a lower-prevalence triage population without recalibration. Among women with abnormal liver tests in pregnancy, AFLP accounted for 5 of 142 cases—about 3.5%—in the prospective Welsh cohort [3]. An illustrative Bayes calculation combining this assumed prevalence with the published sensitivity of 100% and specificity of 57%, under the unverified assumption that sensitivity and specificity remain unchanged across populations, gives a PPV of approximately 8% (Supplementary Table S6). This is a hypothetical value, not an observed PPV in any contemporary clinical population. The reported NPV of 100% follows from the reported sensitivity of 100%, which implies no false negatives, rather than from the high prevalence; it rests on four true negatives, and so small a sample does not establish that a score below six excludes early AFLP.

Two further features limit inference. Every patient was ill enough to warrant biopsy or had died, a severe-spectrum population in which a composite weighted towards features of advanced disease would be expected to perform well. And, the comparator was not healthy pregnancy but other pregnancy-related liver disease—the clinically relevant comparison, and the one in which a specificity of 57% is most damaging.

### 3.2. Specificity Against the Principal Mimics

Reports from other populations support this concern, although they describe criterion fulfilment in women with other conditions rather than formal estimates of specificity. Devarbhavi and colleagues applied the Swansea criteria across AFLP, HELLP syndrome, pre-eclampsia and viral acute liver failure in pregnancy, and concluded that HELLP syndrome and virally mediated acute liver failure also meet the criteria, which in their view, merits reappraisal and re-evaluation of the criteria [25]. This matters most where viral hepatitis is common. Across 47 studies and 3968 women from African and Asian populations, hepatitis E in pregnancy carried a pooled maternal case-fatality risk of 20.8% (95% CI 16.6–25.3), rising to 61.2% among those with fulminant hepatic failure [26]. In a New Delhi cohort of 220 pregnant women with acute viral hepatitis, hepatitis E was associated with fulminant hepatic failure (relative risk [RR] 2.7, 95% CI 1.7–4.2) and with maternal death (RR 6.0, 95% CI 2.7–13.3) [27]. A woman with hepatitis E-related acute liver failure in the third trimester will have vomiting, abdominal pain, jaundice, coagulopathy, encephalopathy, renal impairment and leucocytosis. She satisfies the Swansea criteria comfortably, and the phrase “in the absence of another explanation” is doing most of the diagnostic work.

Several other conditions behave the same way. In herpes simplex hepatitis, fever was the initial symptom in all 24 reported pregnancies, six of which ended in maternal death [28]. Pregnancy-associated thrombotic thrombocytopenic purpura is identified by an activity of a disintegrin and metalloproteinase with a thrombospondin type 1 motif, member 13 (ADAMTS13) below 10%, and is separated from complement-mediated thrombotic microangiopathy by prominent neurological rather than severe renal involvement; AFLP is suggested instead by hypoglycaemia, hyperammonaemia, coagulopathy of hepatic synthetic failure and rapid improvement after delivery [29]. Haemophagocytic lymphohistiocytosis mimics both HELLP syndrome and AFLP closely [30,31]; among the pregnancies with available data in a review of 81 cases, maternal mortality was 22% and foetal mortality 40% [30]. And, paracetamol hepatotoxicity accounted for 21 of the 35 non-pregnancy-specific cases of acute liver injury or failure in pregnancy identified over two decades by the United States Acute Liver Failure Study Group [32]. That multicentre registry, which included no cases of hepatitis A–E, supplies the most direct primary evidence on the point: among 70 women with acute liver injury or failure in pregnancy, the Swansea criteria were met not only by every woman with pregnancy-associated liver disease but also, in the investigators’ words, by “virtually all women with other forms of ALF [acute liver failure]” [32]. Discriminating features for each are summarised in Supplementary Table S3.

### 3.3. What Has Not Yet Been Measured

To our knowledge, no study has applied the Swansea criteria prospectively, at presentation, to a consecutive and undifferentiated population of women with new hepatic dysfunction after 20 weeks of gestation, with an independently adjudicated reference standard. Every estimate of performance that we identified comes either from a severe-spectrum biopsy series [22] or from cohorts assembled because the women already carried the diagnosis. The United Kingdom surveillance study reported that, of the cases notified to the system, 55 (90%) were confirmed by diagnostic criteria together with clinical assessment and two (3%) by clinical assessment alone, giving 97% agreement and a kappa statistic of 0.78 [1]. That is a measure of concordance between two ways of labelling women notified as possible AFLP, not an estimate of specificity. A systematic review of second-trimester AFLP found that 13 of 14 women met the criteria and 10 of 11 biopsied had microvesicular steatosis [33]—again reassuring about criterion fulfilment in confirmed cases, and silent about false positives.

In consequence, fulfilment rates in cohorts that define AFLP by the criteria themselves are not unbiased estimates of sensitivity; specificity has been characterised only in severe-spectrum populations, where it appears limited; and predictive values in the population in which the criteria are actually deployed remain unmeasured.

## 4. The Timing Problem: A Hypothesis of Criterion Accumulation

### 4.1. Retrospective Observations on Criterion Fulfilment Before the Final Diagnosis

The most direct observation on criterion fulfilment before the final diagnosis comes from a retrospective Chinese case–control study designed to find an earlier diagnostic test. Zhong and colleagues compared 41 women with AFLP against 172 controls selected by diagnosis from 28,800 records: 40 with gastroenteritis, 11 with severe viral hepatitis, 40 with HELLP syndrome, 41 with intrahepatic cholestasis of pregnancy and 40 with cholestasis plus pre-eclampsia. Laboratory values were taken approximately three days before the final Swansea-based diagnosis; that timepoint was defined relative to the final diagnosis and does not necessarily correspond to first clinical presentation. At that earlier timepoint, none of the 41 women with AFLP met six criteria; 19.5% met five, 75.6% met four and 4.9% met three [34]. The item-level data show how narrowly the threshold was missed: at that timepoint, all 41 women had an aminotransferase above 42 IU/L and a total bilirubin above 0.8 mg/dL, 40 (97.6%) a prothrombin time above 14 s, 39 (95.1%) an activated partial thromboplastin time above 34 s and 39 (95.1%) a leucocytosis [34]. A combination of gastrointestinal symptoms with aminotransferase, bilirubin, bile acid and clotting-time abnormalities was reported to identify AFLP with 97.6% sensitivity and 97.1% specificity against the Swansea-based final diagnosis, roughly three days before the criteria were satisfied. The authors attributed the delay to the composition of the criteria, noting that “some terms could only be reached when symptoms and signs have progressed, such as encephalopathy, ascites or bright liver on sonogram, which might be too late for treatment” [34].

Several caveats are needed. The control group, though clinically apt, was selected by diagnosis rather than by presenting syndrome, which is likely to inflate specificity relative to a genuine triage population. The reference standard was the Swansea criteria themselves, and because the diagnosis was Swansea-based, failure to meet the threshold before that diagnosis is partly expected by design. The informative finding is therefore that biochemical abnormalities were detectable before fulfilment of the conventional Swansea threshold. The study does not show that these abnormalities identify AFLP with independent accuracy, it has not been validated in an undifferentiated triage population, and it does not establish a validated early diagnostic rule.

A different picture emerges when the criteria are applied retrospectively. Among 73 women with a clinical diagnosis of AFLP, identified from 24,054 obstetric and intensive care admissions at a single Beijing hospital, retrospective rescoring showed that 66 (90.4%) satisfied six or more criteria and 7 (9.6%) did not [13]. The two observations are not directly comparable—they come from different cohorts, sampled at different points in the illness and assessed by different methods—but together, they are consistent with a composite that is frequently fulfilled once the disease is clinically established and less often fulfilled earlier in its course. Because the two cohorts are separate, these data do not demonstrate within-patient progression; the sequence they suggest is a hypothesis that requires prospective longitudinal evaluation.

### 4.2. Criterion Accumulation and Prognosis

If the criterion count tracks disease intensity, it should carry prognostic information, and limited retrospective data suggest that it does. In the same Beijing cohort, the Swansea score discriminated hepatic encephalopathy (area under the receiver operating characteristic curve [AUC] 0.773, optimal cut-off 8) and acute renal failure (AUC 0.796, cut-off 8). It also discriminated the need for continuous renal replacement therapy (AUC 0.805, cut-off 8) and stillbirth (AUC 0.894, cut-off 10) [13]. Across the eleven complications and treatment requirements reported, AUCs ranged from 0.694 to 0.894 and the optimal cut-offs from 7 to 10, one to four points above the diagnostic threshold; these cut-offs were derived within the same retrospective sample and have not been validated. The guideline of the International Federation of Gynecology and Obstetrics (FIGO) likewise lists more than seven Swansea criteria, alongside a raised serum lactate and a Model for End-stage Liver Disease score of 30 or more, among the features predicting the need for intensive care [12]. In a liver-unit series of severe pregnancy-related liver disease, a raised admission lactate predicted death or liver transplantation with 73% sensitivity and 75% specificity [35]. Both sources print the lactate threshold as 2.8 mg/dL, which would correspond to approximately 0.3 mmol/L and is physiologically implausible; no correction to either source was identified, the unit discrepancy could not be resolved and the numerical threshold is therefore not used for interpretation in this review. And, in a Swiss series of six women with AFLP, the cumulative number of Swansea criteria correlated strongly with serum soluble fms-like tyrosine kinase-1 (sFlt-1) concentration (r = 0.97, *p* < 0.01), with the highest values in women meeting eight or more criteria [36].

**Figure 1:**
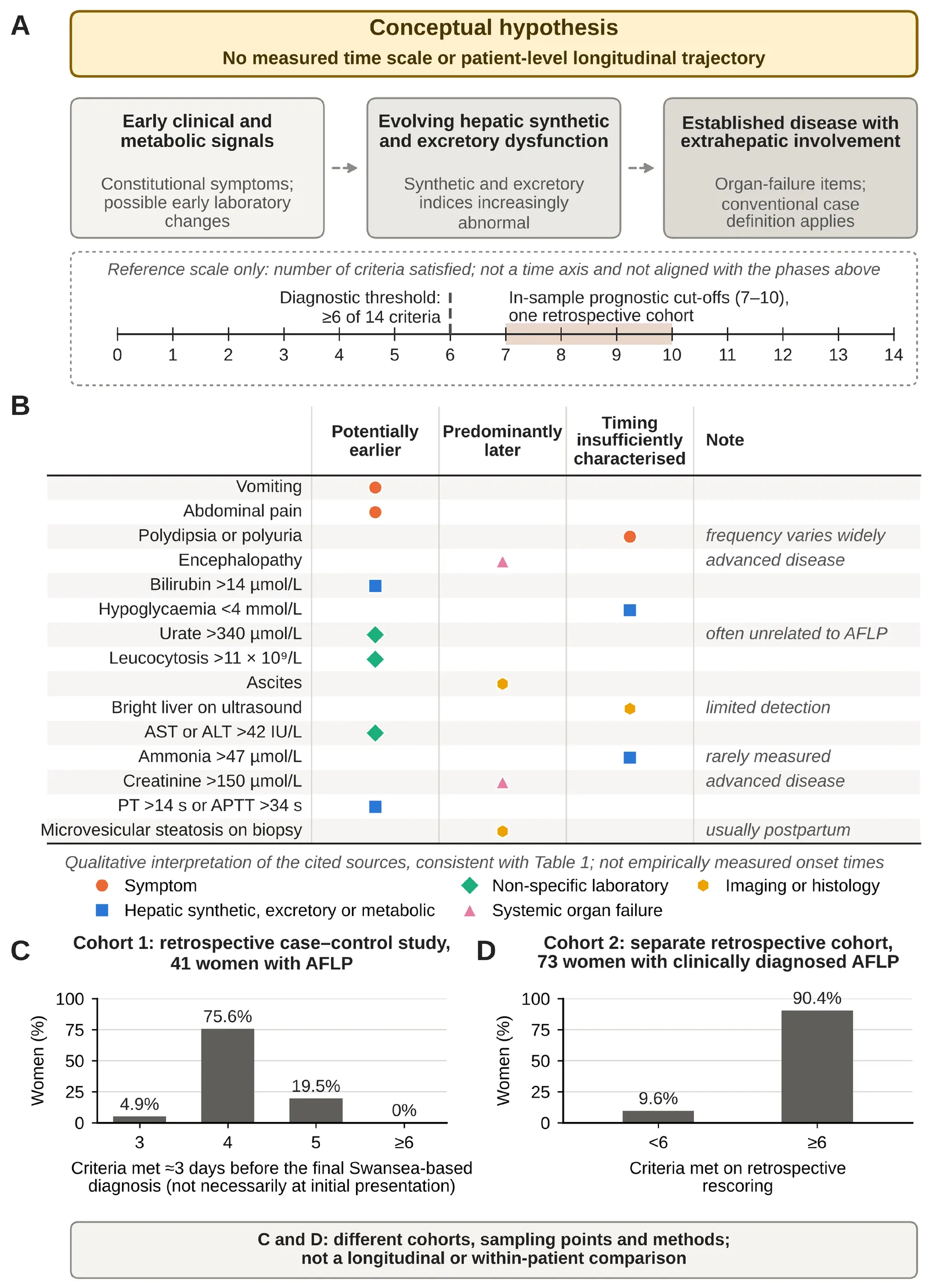
Conceptual interpretation of Swansea criterion fulfilment in acute fatty liver of pregnancy (AFLP). (**A**) Hypothesised sequence of recognition phases, from early clinical and metabolic signals, through evolving hepatic synthetic and excretory dysfunction, to established disease with extrahepatic involvement. The schematic is a conceptual hypothesis with no measured time scale; it is not derived from patient-level longitudinal data and does not depict criterion counts over time. The criterion-count scale beneath it is not aligned with the phases and shows, for reference only, the diagnostic threshold of six criteria and the range of counts (7–10) at which one retrospective cohort reported in-sample optimal cut-offs for maternal and perinatal complications and treatment requirements [13]. (**B**) Qualitative interpretation of when each criterion may become satisfiable, consistent with Table 1, with markers coloured and shaped by domain (the hepatic synthetic, excretory and metabolic domains of Table 1 are combined, as are imaging and histology); the two components of the imaging item are shown separately. These assignments are qualitative interpretations of the cited sources, not empirically measured onset times. (**C**) Published distribution of criterion counts in 41 women with AFLP, based on measurements obtained approximately three days before the final Swansea-based diagnosis, not necessarily at initial presentation, in a retrospective case–control study [34]. (**D**) Published proportions of 73 women with a clinical diagnosis of AFLP who did and did not meet the threshold when the criteria were applied retrospectively in a separate cohort [13]. (**C**,**D**) come from different cohorts, sampled at different points in the illness and assessed by different methods; they do not establish within-patient progression. The figure was drawn de novo from the cited data. ALT, alanine aminotransferase; APTT, activated partial thromboplastin time; AST, aspartate aminotransferase; PT, prothrombin time.

These findings suggest that the composite captures clinically meaningful disease intensity, with the prognostically informative range lying above the diagnostic threshold, although the criteria have not been developed or validated as a severity instrument. Figure 1 presents this interpretation as a conceptual schematic, alongside the published distributions of criterion counts in the two cohorts described above. Table 1 sets out, item by item, a qualitative interpretation of when each criterion may become satisfiable.

### 4.3. Does Delay Actually Matter?

The clinical case for earlier diagnosis rests on associations rather than trial evidence, and this should be stated plainly. In a four-centre Chinese cohort of 133 women, 13 (9.8%) were diagnosed only after delivery, and postpartum diagnosis was associated with worse maternal outcome in univariable analysis (*p* < 0.01) [14]. In a two-centre cohort of 290, the interval from symptom onset to hospitalisation and from symptom onset to delivery were both associated with death [6]. In an earlier Taiwanese series, 12 of 18 women (67%) were diagnosed only postpartum, with two maternal deaths [44]. In the study that applied a diagnostic model approximately three days before criteria fulfilment, maternal mortality was lower and Apgar scores higher in the 23 women whose delivery was prompted by the model than in the 18 delivered after a Swansea-based diagnosis, a non-randomised comparison in which the model results were disclosed to the treating clinicians [34]. The ACG guideline states that the postpartum course depends on the interval between symptoms and termination of pregnancy [11], and up to 20% of cases are reported to present after delivery [19].

Most women nonetheless recover with delivery and supportive care [7,45], which is what makes the timing of recognition potentially consequential. All of these associations are vulnerable to reverse causation and confounding by severity: women whose illness declares itself abruptly are both more likely to be diagnosed late and more likely to die. To our knowledge, no randomised or quasi-experimental evidence establishes that earlier recognition improves maternal survival, and none is likely to be generated for a disease of this rarity. The defensible position is narrower. Earlier recognition is mechanistically plausible and has been associated with better outcome in several observational series, and it changes several decisions irrespective of any effect on mortality. Those decisions include the urgency of delivery, and correction of hypoglycaemia and coagulopathy before operative delivery and before any neuraxial technique. They include the acceptability of any delay to delivery: a brief delay for antenatal corticosteroids may be considered in selected stable women with HELLP syndrome remote from term, whereas expectant management is not appropriate in AFLP [11,12]. They also include early referral to a transplant-capable centre [12,46], the intensity of organ support [6,47], screening of the neonate for fatty acid oxidation disorders [11,48] and counselling about recurrence [49].

### 4.4. The Prodrome Is Real but Non-Specific

The presenting symptoms reported in AFLP cohorts are largely constitutional. Persistent nausea and vomiting were present in 57% of 51 women at Parkland Hospital, hypertension in 57% and abdominal pain in 53%, with more than 90% having at least one [7]; gastrointestinal manifestations occurred in 87.8% of pooled cases in a systematic review [48], and nausea and vomiting in 87.7% of 73 women in a recent retrospective series [50]. Reported frequencies nonetheless vary widely, from jaundice in all 32 women of one South Chinese series [51] to anorexia in 44.4% of 133 in another [5]. That spread probably reflects differences in symptom ascertainment, and in the point of the illness at which each cohort was assembled, rather than genuine biological heterogeneity.

Two symptoms deserve specific comment. Polydipsia was noted in nine of twelve episodes in the classical Chilean series [37], whereas polydipsia or polyuria was recorded in only 9.4% of a modern cohort [38]. Transient diabetes insipidus has been described in six women, all with biopsy-proven AFLP; desmopressin reduced urine output in each of the five to whom it was given, and symptoms resolved by the end of the fourth postpartum week [39]. Thirst in a jaundiced third-trimester woman is an unusual complaint with a short differential. Fever, by contrast, is not a Swansea item and can actively mislead: in an Indonesian series, it “served to deceive and disorient the first treating clinician, resulting in a late referral” [52]. AFLP can also present with minimal symptoms and be recognised through laboratory abnormalities, with confirmation on imaging [53], which is a further argument for a laboratory-anchored rather than symptom-anchored early construct.

## 5. From Mechanism to Measurable Early Signal

### 5.1. Foetal Fatty Acid Oxidation Defects: Demonstrable in a Minority of Tested Pregnancies

The dominant pathogenic model derives from a small number of mother–infant observations reported in the early 1990s. In one series, every pregnancy carrying an infant later found to have long-chain 3-hydroxyacyl-coenzyme A dehydrogenase (LCHAD) deficiency was complicated by features such as fatty liver or HELLP syndrome, whereas pregnancies with unaffected infants were largely uncomplicated [54]. In the most detailed index case, a woman with AFLP gave birth to an apparently normal infant who presented at four months of age with hypoglycaemia, coma and profound hepatic steatosis; the infant had LCHAD deficiency and the mother proved to be a heterozygous carrier [55]. Molecular characterisation followed. LCHAD is one of three enzyme activities of the mitochondrial trifunctional protein (MTP), together with long-chain enoyl-coenzyme A hydratase and long-chain 3-ketoacyl-coenzyme A thiolase; isolated LCHAD deficiency denotes selective loss of the LCHAD activity, whereas complete MTP deficiency denotes loss of all three [56,57]. The common variant in isolated LCHAD deficiency was reported as a G1528C transversion that substitutes glutamine for glutamic acid at residue 474 of the mature α-subunit [56] and was designated E474Q (Glu474Gln) in subsequent studies [57]; in current nomenclature, it is c.1528G>C (p.Glu510Gln) in HADHA, the gene encoding the MTP α-subunit (reference sequence NM_000182.5), with residues numbered on the precursor protein that includes the mitochondrial targeting sequence. The E474Q designation is retained below when those studies are described. In the pivotal series, 79% of heterozygous mothers carrying a foetus with the E474Q variant developed AFLP or HELLP syndrome [57]. Molecular screening of 108 consecutive referred samples subsequently detected variants causing LCHAD deficiency in five families (19%) among 27 pregnancies complicated by AFLP, while only one of 81 women with HELLP syndrome was heterozygous and no variant was found in her newborn [58]. A matched case–control study extended the association beyond long-chain defects. Maternal liver disease occurred in 16.0% of pregnancies carrying a foetus with any fatty acid oxidation defect versus 0.88% in the general population (odds ratio 20.4, 95% CI 7.82–53.2), with a roughly 50-fold excess for long-chain and 12-fold for short- and medium-chain defects [59]. A recent systematic review of 77 AFLP pregnancies that underwent genetic or biochemical testing found a confirmed foetal defect in 27 (35.1%), of which 22 (81.5%) were LCHAD deficiency [48]; because these pregnancies were selected for testing, this proportion is not a population prevalence.

The same evidence sets the limits of the model, and these are frequently understated. Roughly two-thirds of tested AFLP pregnancies have no demonstrable foetal defect [48,58], while 84% of pregnancies carrying an affected foetus produce no maternal liver disease at all [59]. Within the original series, five children with complete MTP deficiency carried no E474Q allele, and none of their mothers had liver disease in pregnancy [57]. The carrier frequency of the c.1528G>C variant in the Netherlands is approximately 1 in 680, and heterozygosity is not a major contributor to HELLP syndrome [60]. The E474Q variant was not detected in 103 women with HELLP syndrome, 82 of their children and 103 controls [61]. Foetal fatty acid oxidation defects are therefore a well-established association in a subset of AFLP pregnancies selected for testing, although such defects are not demonstrable in all tested AFLP pregnancies and maternal liver disease does not invariably occur when the foetus is affected. Its clinical implication is postnatal rather than diagnostic: guidance recommends molecular testing of mother and child and surveillance of the infant for hypoketotic hypoglycaemia [11,12]. Supplementary Table S4 collates the evidence for this association.

### 5.2. Microvesicular Steatosis as a Predominantly Functional Lesion: Implications for Which Markers May Change Early

Whatever initiates it, the hepatic lesion is distinctive. In the prevailing model, 3-hydroxy fatty acids and 3-hydroxydicarboxylic acids accumulate in the placenta and reach the maternal circulation, where they are proposed to be lipotoxic through oxidative stress, mitochondrial dysfunction and hepatocyte lipoapoptosis [62]. The placental and mitochondrial limbs of that model rest on human placental mitochondrial studies and on murine MTP knockouts; the hepatocyte lipoapoptosis limb remains a hypothesis rather than a demonstrated step [62]. The evidence for mitochondrial dysfunction and placental injury in AFLP, including placental lipid accumulation, has been reviewed in detail [63]. Human histological and ultrastructural observations are consistent: megamitochondria with paracrystalline inclusions were demonstrated in four specimens in the Chilean series [37], and a recent biopsy-proven case showed microvesicular steatosis predominantly around the central veins, with abundant cytoplasmic lipid droplets on electron microscopy [23]. Reviews of the histology describe microvesicular fat within swollen hepatocytes with little or no inflammation, frequent periportal sparing and necrosis that is usually limited, being more evident in severe cases [10,19].

The clinically important corollary is that hepatocyte function in AFLP is impaired out of proportion to structural destruction. Unlike the confluent necrosis of viral or drug-induced hepatitis, microvesicular steatosis is thought to produce a predominantly bioenergetic failure: loss of adenosine triphosphate-dependent synthetic capacity, of biliary excretion and of gluconeogenesis, with comparatively modest cytolysis. The predicted biochemical signature is therefore synthetic and excretory failure disproportionate to transaminase elevation, and the published cohorts are consistent with it. In 133 women, mean alanine aminotransferase was 287 ± 280 U/L and aspartate aminotransferase 254 ± 246 U/L. Against those figures stood a mean prothrombin time of 16.7 ± 7.5 s and a fibrinogen of 1.7 ± 1.0 g/L [5]. Comparable values are reported elsewhere [38].

**Figure 2:**
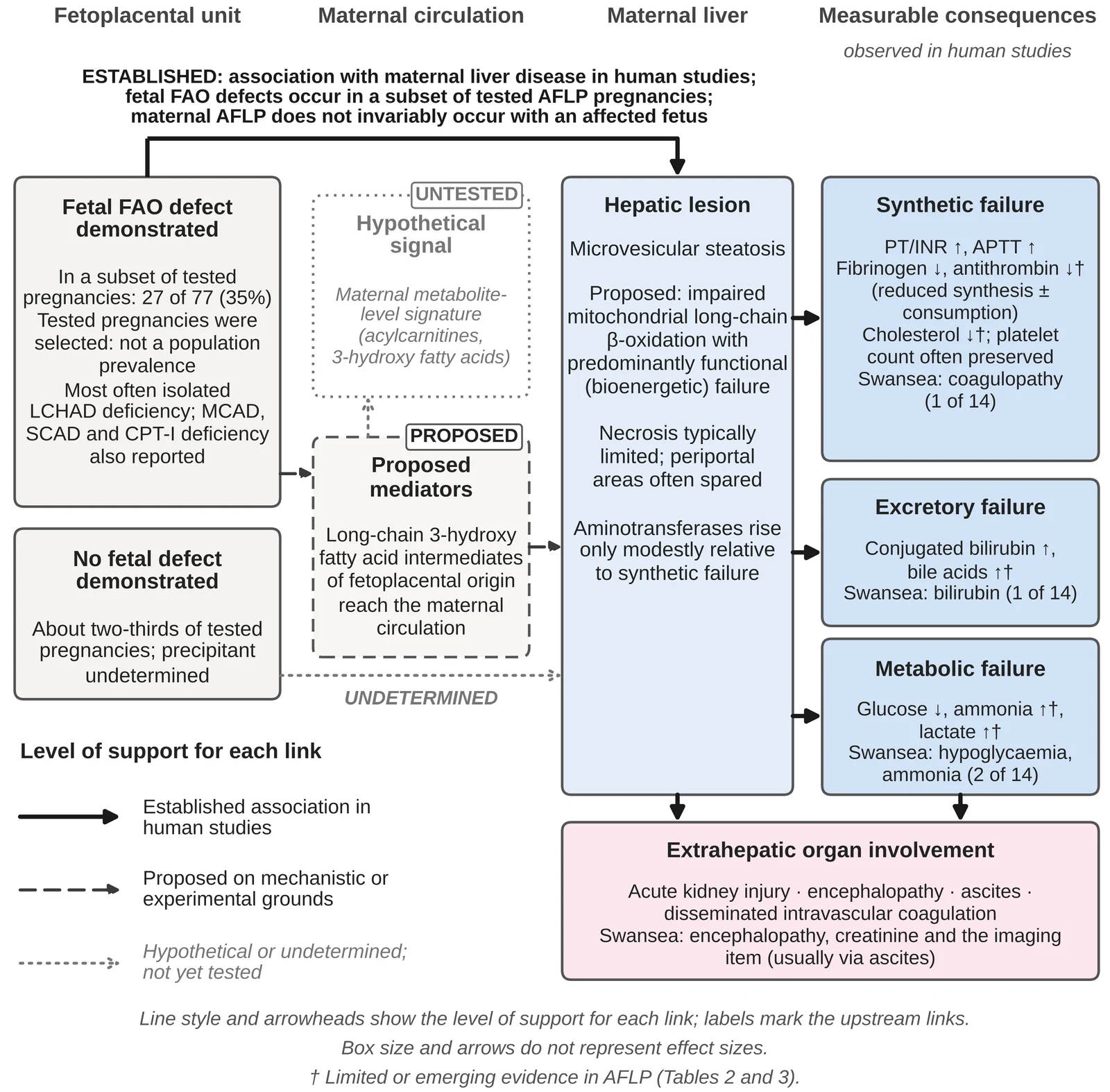
Proposed mechanism of hepatic injury in acute fatty liver of pregnancy (AFLP), its measurable consequences and the Swansea criteria that index each. The level of support for each link is indicated by line style and, for the upstream links, by an explicit label: solid lines with filled arrowheads, associations established in human studies; dashed lines, links proposed on mechanistic or experimental grounds; dotted lines with open arrowheads, hypothetical or undetermined links not yet tested. Hypothetical elements are drawn with dotted, unfilled outlines so that they do not appear equivalent to established findings. A foetal fatty acid oxidation (FAO) defect, most often isolated long-chain 3-hydroxyacyl-coenzyme A dehydrogenase (LCHAD) deficiency, has been confirmed in only a subset of tested pregnancies (27 of 77 in a recent systematic review [48]); these pregnancies were selected for testing, so this proportion is not a population prevalence, and in the remaining pregnancies, no foetal defect was demonstrated and the precipitant is undetermined. Long-chain 3-hydroxy fatty acid intermediates of foetoplacental origin are the proposed circulating mediators [62,63]. The hepatic lesion is a microvesicular steatosis with typically limited necrosis, thought to reflect impaired mitochondrial long-chain β-oxidation with predominantly functional, bioenergetic failure [10,19]; it produces synthetic, excretory and metabolic failure disproportionate to the rise in aminotransferases, and fibrinogen and antithrombin fall through reduced synthesis, with a variable contribution from consumption. Consequences marked † are those for which the evidence in AFLP is limited or emerging (Table 2 and Table 3). The markers within the three hepatocellular domains are not expected to become abnormal simultaneously; Figure 1 and Table 1 give a qualitative interpretation of item-level timing. Of the fourteen Swansea criteria, one addresses hepatic synthetic function, one excretory function and two metabolic function; encephalopathy and creatinine index extrahepatic organ involvement, and the imaging item combines an ultrasound finding with limited detection in the available AFLP series and ascites, a typically later feature. A maternal metabolite-level signature is shown as a hypothetical, untested signal. The figure was drawn de novo as a synthesis of the cited sources. APTT, activated partial thromboplastin time; CPT-I, carnitine palmitoyltransferase I; INR, international normalised ratio; MCAD, medium-chain acyl-coenzyme A dehydrogenase; PT, prothrombin time; SCAD, short-chain acyl-coenzyme A dehydrogenase.

The Swansea transaminase threshold of 42 IU/L is set so low that almost every woman with any hepatic disturbance in pregnancy will meet it. It contributes to criterion fulfilment but little to discrimination. The informative signal is probably not the height of the transaminases but the disproportion between them and the synthetic indices. Figure 2 maps the proposed mechanism onto its measurable consequences and onto the Swansea items that index each, distinguishing established, proposed and hypothetical links, and makes visible how few criteria address the synthetic axis.

### 5.3. Metabolite-Level Signals of Fatty Acid Oxidation Failure

Because the primary lesion is thought to involve impaired mitochondrial long-chain fatty acid β-oxidation, AFLP could plausibly generate a metabolite-level signature that may precede some downstream laboratory abnormalities; this is a hypothesis that warrants prospective evaluation. In the affected infant, isolated LCHAD deficiency and complete MTP deficiency produce diagnostic elevations on plasma acylcarnitine analysis, and long-chain 3-hydroxydicarboxylic acids may appear in urinary organic acids; most fatty acid oxidation disorders are detectable by newborn screening with tandem mass spectrometry, although programmes differ between countries, and the infants of affected pregnancies should be screened at birth [64]. Neonatal testing is therefore an established diagnostic application. On the maternal side, increased circulating long-chain and 3-hydroxy fatty acids have been described in women with established AFLP (reviewed in [62,63]); measurements made in established disease do not indicate early diagnostic performance. To our knowledge, the timing of these metabolites relative to clinical diagnosis and their performance against the principal mimics have not been evaluated, maternal acylcarnitine profiles have not been systematically reported, and untargeted metabolomic or lipidomic profiling of maternal blood in AFLP remains largely unexplored, so maternal diagnostic applications remain hypothetical.

The downstream abnormalities already discussed can also be read as metabolic readouts of hepatic energy failure rather than as isolated laboratory findings: hypoglycaemia reflects failed gluconeogenesis, hyperammonaemia failed ureagenesis, hyperlactataemia reduced hepatic lactate clearance, hypocholesterolaemia impaired hepatic lipoprotein synthesis, and raised bile acids impaired canalicular transport (Figure 2 and Table 2; Section 6.4 and Section 6.5). This framing has two implications for research. First, a targeted panel comprising the acylcarnitine profile, 3-hydroxy fatty acids, the bile acid profile and lipoprotein fractions could be measured at presentation alongside conventional indices (Section 9). Second, because maternal complications have been associated more strongly with an affected foetal genotype than with maternal carrier status [54,57], a maternal metabolic signature, if one exists, would be expected to reflect the foetoplacental and hepatic state of the index pregnancy rather than a stable maternal trait, supporting investigation of serial rather than single measurements.

### 5.4. A Second, Partly Independent Axis: The Placental Angiogenic Signature

Anti-angiogenic factor release provides a biologically distinct signal. In a Swiss cohort, sFlt-1 was markedly higher in AFLP than in HELLP syndrome (median 57,570 pg/mL, range 31,609–147,170, versus 9713 pg/mL, range 1348–30,781; *p* < 0.001). Placental growth factor (PlGF) was higher as well (197 versus 40 pg/mL; *p* < 0.01). That is the opposite of the pre-eclampsia pattern, and it explains why the sFlt-1/PlGF ratio did not differ between the groups [36]. An independent Dutch group reported concordant findings. In an initial series of 11 women, sFlt-1 was higher in singleton AFLP pregnancies than in suspected or confirmed pre-eclampsia [65]. In a subsequent analysis, the median sFlt-1 was 77,762 pg/mL in 12 antepartum AFLP cases against 14,572 pg/mL in HELLP syndrome, and the median total PlGF was 2054 pg/mL in AFLP against 354, 435 and 344 pg/mL in women without pre-eclampsia, with pre-eclampsia and with HELLP syndrome, respectively [66]. Total PlGF fell after delivery, and in non-pregnant acute liver failure, sFlt-1 was raised (median 446 pg/mL) but far below antepartum AFLP values, while total PlGF was normal; the authors interpret this as indicating a placental rather than hepatic origin of the PlGF elevation [66]. Two small cohorts from different countries agreeing on an unexpected direction of effect is a stronger signal than either alone, and it is compatible with a placental stress response in AFLP that differs qualitatively from that of pre-eclampsia, although this remains to be confirmed.

## 6. Candidate Early Discriminators: What the Evidence Actually Supports

Table 2 summarises the appraisal that follows, distinguishing biological rationale, association with AFLP, diagnostic accuracy, prognostic value and clinical utility. None of the candidates has a validated early diagnostic threshold, and clinical utility, an effect of marker use on management or outcome, has not been demonstrated for any of them.

**Table 2:** Candidate markers for earlier recognition of acute fatty liver of pregnancy: biological rationale; association and diagnostic accuracy against comparators; prognostic evidence within AFLP; limitations, threshold status and clinical utility; and certainty.

Candidate Marker	Biological Rationale	Association and Diagnostic Accuracy (AFLP Versus Comparators) ^b^	Prognostic Evidence (Within AFLP) ^c^	Limitations, Threshold Status and Clinical Utility ^d^	Certainty ^a^
Prothrombin time, INR, APTT	Short-lived clotting factors fall early when hepatic synthesis fails; pregnancy shortens clotting times, so any prolongation is conspicuous	PT 14.1 versus 9.5 s and APTT 38.5 versus 27.9 s versus HELLP syndrome (both *p* < 0.001; *n* = 21 versus 80) [40]; PT > 14 s in 40 of 41 cases versus 7 of 172 diagnosis-selected controls approximately three days before the final diagnosis [34]; cases only: pooled median PT-INR 1.59 (IQR 1.31–2.02) in a case-report series of 102 patients [42]	PT and INR AUC 0.837 for a composite adverse outcome that included coagulopathy (*n* = 92) [4]; PT AUC 0.77 for progression to acute liver failure (*n* = 133) [5]	Case–control and severe-spectrum samples only; prospective validation at initial presentation remains an evidence gap; thresholds based on non-pregnant reference intervals; no validated early-diagnostic threshold	Association: Probable; early diagnostic use: Emerging
Fibrinogen	Physiologically elevated in pregnancy, so a “normal” value is already abnormal; falls through reduced synthesis ± consumption	193 versus 408.1 mg/dL versus HELLP syndrome (*p* < 0.001) [40]; proposed as one of four admission discriminators [67]; cases only: pooled median 82 mg/dL (IQR 52.5–153.5) [42]	AUC 0.320 (95% CI 0.208–0.432; *p* = 0.004) for a composite adverse outcome, i.e., lower values associated with the outcome (*n* = 92) [4]	Falls in abruption, sepsis and any disseminated intravascular coagulation; no diagnostic threshold derived for pregnancy	Association: Probable; early diagnostic use: Knowledge gap
Antithrombin activity	Predominantly hepatic synthesis; falls through reduced synthesis ± consumption	Cases only: severe depression in the earliest series [68]; pooled median 23.0% (IQR 17.0–33.0) in a case-report series of 102 patients [42]; low activity described as distinguishing AFLP from HELLP syndrome in a small comparative series [69]	AUC 0.420 (95% CI 0.294–0.546; *p* = 0.202) for a composite adverse outcome (*n* = 92) [4]; this prognostic result does not address diagnostic value	Threshold of <65% proposed [43] but neither derived from a diagnostic-accuracy analysis nor validated; 87% in one biopsy-proven case [23]; not measured routinely in all settings	Association: Emerging; early diagnostic use and threshold: Knowledge gap
Platelet count	AFLP is primarily a synthetic failure rather than a consumptive microangiopathy	174 versus 89 × 10^3^/µL versus HELLP syndrome [40]; cases only: approximately 50% not thrombocytopenic at presentation [43]; pooled median 16.1 × 10^4^/µL, with ~70% ≥ 12 × 10^4^/µL [42]	—	Low platelet counts do occur, particularly with established disseminated intravascular coagulation, so their presence does not exclude AFLP	Association (higher count than in HELLP syndrome): Probable; early diagnostic use in isolation: Knowledge gap
Conjugated bilirubin	Failure of canalicular excretion by energy-depleted hepatocytes	Total bilirubin 4.4 versus 0.9 mg/dL versus HELLP syndrome [40]; proposed admission discriminator [67]; cases only: conjugated fraction 62–85% of total (*n* = 106 and 133) [5,38]; total bilirubin above 0.8 mg/dL in all 41 cases approximately three days before the final diagnosis, as a component of the only pre-threshold model identified [34]	Total bilirubin AUC 0.80 (95% CI 0.72–0.88) for progression to acute liver failure (*n* = 133) [5]; independent predictor of maternal death (odds ratio 1.009, 95% CI 1.003–1.014) [14]	Swansea threshold of 14 µmol/L too low to discriminate; no threshold derived for early diagnosis	Association: Probable; early diagnostic use: Emerging
Total bile acids	Cholestatic component of the hepatic lesion	Above 10 µmol/L in 41 of 41 cases versus 88 of 172 diagnosis-selected controls approximately three days before the final diagnosis; component of the only pre-threshold model identified [34]	—	Markedly raised in intrahepatic cholestasis of pregnancy, the commonest competing diagnosis; single study; no validated threshold	Emerging (single study)
Blood glucose	Failure of hepatic gluconeogenesis has few competing explanations in late pregnancy	85.0 versus 98.5 mg/dL versus HELLP syndrome (*p* < 0.001) [40]; below 72 mg/dL in 5 of 41 cases (12.2%) versus 3 of 172 controls approximately three days before the final diagnosis [34]; cases only: hypoglycaemia in 76 of 133 (57.1%) once established [14]	—	Insensitive earlier in the illness: adding glucose to the diagnostic model reduced sensitivity from 97.6% to 12.2% [34]; mean glucose within the reference interval in a cohort of 106, sampling time unspecified [38]	Association: Probable; low sensitivity earlier in the illness: Emerging
Total and LDL cholesterol, triglycerides	Impaired hepatic lipoprotein synthesis, against a physiological rise in pregnancy	Low cholesterol and triglycerides described as distinguishing AFLP from HELLP syndrome [69]; cholesterol among four proposed admission discriminators [67]; hypocholesterolaemia in a case of presumed AFLP [70]	—	Small numbers, inconsistent sampling times, no derived threshold, rarely measured in practice; a reported difference in LDL cholesterol is uninterpretable because the study’s cases had normal liver tests and no stated case definition [71]	Emerging
Soluble fms-like tyrosine kinase-1 (±PlGF)	Placental stress response distinct from that of pre-eclampsia, and partly independent of the hepatic synthetic axis	Association: median 57,570 versus 9713 pg/mL versus HELLP syndrome (*p* < 0.001) [36], with elevated concentrations replicated by an independent group (median 77,762 pg/mL in 12 antepartum cases, with markedly elevated total PlGF) [65,66]. Accuracy (in-sample): cut-off of 31,100 pg/mL with 100% sensitivity and specificity, derived in six AFLP cases and not externally validated [36]	Correlated with the number of Swansea criteria met (r = 0.97; six cases) [36]	Very small samples; threshold optimally chosen in the derivation sample and not externally validated; replication supports the direction of the association, not the cut-off; limited availability and turnaround	Association (direction): Probable; threshold: Emerging
Ultrasound, computed tomography, magnetic resonance imaging	Direct detection of hepatic fat	Fatty change in 3 of 11 by ultrasound, 5 of 10 by computed tomography and 0 of 5 by magnetic resonance imaging in the classical radiological series [41]; ultrasonographic fatty change in 20 of 133 [5]; abnormal ultrasound in 4 of 41 cases versus 12 of 172 controls approximately three days before the final diagnosis [34]	—	Limited detection of hepatic fat in the available AFLP series; detection of fat did not alter management in the classical series [41]	Established (limited detection)
Controlled attenuation parameter, transient elastography	Quantification of hepatic steatosis and stiffness	Single biopsy-proven case: controlled attenuation parameter 196 dB/m (grade S0) despite imaging and histological steatosis; liver stiffness 11.2 kPa [23]	—	Attenuation-based methods may underestimate microvesicular fat; no series data	Knowledge gap
Portal vein Doppler velocity	Proposed sinusoidal compression by swollen, lipid-laden hepatocytes	Association: 22.9 versus 27.7 cm/s versus controls. Discrimination without a stated reference standard: nomogram AUC 0.88 (95% CI 0.82–0.95) in derivation and 0.86 (0.72–0.98) in an internal test set [71]	—	‘AFLP’ group recruited from healthy antenatal attendees, with normal liver tests and no stated case definition; single centre, internal split only, operator dependent	Knowledge gap
Ammonia and lactate	Loss of ureagenesis and of hepatic lactate clearance	Ammonia did not differ from HELLP syndrome in the one comparative study that reported it [40]	Raised lactate listed as a predictor of intensive care admission [12] and, in a liver-unit series, of death or transplantation [35]; the threshold printed in both sources (2.8 mg/dL) carries an unresolved unit discrepancy and is not used for interpretation	Rarely reported in modern AFLP cohorts; distributions in AFLP poorly described; no diagnostic threshold	Knowledge gap
Acylcarnitines and 3-hydroxy fatty acids	Direct readouts of impaired long-chain β-oxidation; foetoplacental 3-hydroxy fatty acids are the proposed circulating mediators	Neonatal: diagnostic acylcarnitine elevations in affected infants, detectable by newborn screening [64]. Maternal: increased long-chain and 3-hydroxy fatty acids described in established AFLP [62,63]; no diagnostic-accuracy data	—	No maternal data on timing, performance against mimics or thresholds; maternal carrier status alone is not diagnostic	Neonatal testing: Established; maternal diagnostic use: Knowledge gap (hypothesis)
Multivariable pre-threshold model	Combines individually weak signals from routine tests	Accuracy (case–control, in-sample): gastrointestinal symptoms with aminotransferase, bilirubin, bile acid and clotting-time abnormalities: sensitivity 97.6%, specificity 97.1% against the Swansea-based final diagnosis, approximately three days before the threshold was met; reported predictive values reflect case–control sampling [34]	Most other models identified are prognostic (Section 6.8)	Single centre, n = 41, controls selected by diagnosis, Swansea criteria as reference standard, no AUC or calibration reported, no external validation; better outcomes after model-prompted delivery derive from a non-randomised comparison and do not establish clinical utility	Emerging

^a^ Qualitative categories assigned by the authors using the criteria described in Section Approach to Literature Identification and Certainty Assessment, not formal GRADE ratings: Established, a well-supported finding with reasonably consistent independent evidence, or a mathematical relationship, applying to the specific statement rather than automatically to early diagnostic usefulness; Probable, credible clinical evidence with important limitations in design, precision, replication or applicability; Emerging, preliminary clinical findings requiring independent confirmation; Knowledge gap, insufficient direct evidence to answer the stated question reliably. Where they differ, the certainty of an association and that of early diagnostic use are given separately. ^b^ Entries describe associations (differences between AFLP and comparator groups) unless labelled as accuracy (sensitivity, specificity or AUC against a stated reference standard); ‘cases only’ denotes descriptive data from women with AFLP without a comparator group, which do not measure discrimination. ^c^ Prediction of outcome among women already diagnosed with AFLP; prognostic performance does not establish diagnostic value. ^d^ Clinical utility, an effect of measuring the marker on management or outcome, has not been demonstrated for any candidate listed. Values are given in the units of the original reports; conversion factors: bilirubin, 1 mg/dL = 17.1 µmol/L; glucose, 1 mmol/L = 18.0 mg/dL; fibrinogen, 1 g/L = 100 mg/dL; platelets, 1 × 10^3^/µL = 1 × 10^9^/L. AFLP, acute fatty liver of pregnancy; APTT, activated partial thromboplastin time; AUC, area under the receiver operating characteristic curve; CI, confidence interval; GRADE, Grading of Recommendations Assessment, Development and Evaluation; HELLP, haemolysis, elevated liver enzymes and low platelets; INR, international normalised ratio; IQR, interquartile range; LDL, low-density lipoprotein; PlGF, placental growth factor; PT, prothrombin time.

### 6.1. Hepatic Synthetic Failure

Pregnancy is a procoagulant state: fibrinogen rises substantially and clotting times shorten. Any prolongation of the prothrombin time, and any fibrinogen concentration that is merely “normal” for a non-pregnant adult, is therefore already abnormal in late gestation. This should give synthetic markers a favourable signal-to-noise ratio, and comparative data from established disease are consistent with it. In a Taiwanese cohort of 21 women with AFLP and 80 with HELLP syndrome, prothrombin time was 14.1 versus 9.5 s, activated partial thromboplastin time 38.5 versus 27.9 s and fibrinogen 193 versus 408.1 mg/dL, all *p* < 0.001. Platelets ran the other way, at 174 versus 89 × 10^3^/µL [40]. The direction of the platelet difference deserves emphasis: in AFLP, the platelet count is often preserved while the clotting is deranged, which is the mirror image of HELLP syndrome. A review of the published literature concluded that approximately 50% of women with AFLP have no thrombocytopenia at presentation [43]. In a pooled analysis of 102 Japanese cases, the median platelet count was within the normal range at 16.1 × 10^4^/µL (interquartile range 11.1–19.2), with roughly 70% at or above 12 × 10^4^/µL [42]. A falling platelet count is therefore an unreliable early signal. Laboratory values reported in the comparative AFLP-versus-HELLP studies are collated in Supplementary Table S2.

By the time of delivery, hepatic synthetic dysfunction is present in the great majority of cases. Procoagulant synthesis was impaired in more than three-quarters of 51 women, half of whom required transfusion [7], and 80% met the International Society on Thrombosis and Haemostasis criteria for unequivocal disseminated intravascular coagulation at delivery, with a mean score of 5.9 ± 1.8 [72]. Whether these abnormalities are demonstrable early enough to be useful rests largely on a single retrospective case–control study: approximately three days before the final Swansea-based diagnosis, a prothrombin time above 14 s was present in 40 of 41 women with AFLP but in only 7 of 172 diagnosis-selected controls [34]. For the reasons given in Section 4.1, these frequencies should not be read as validated estimates of sensitivity or specificity. They are nonetheless the most direct evidence that abnormalities of hepatic synthetic function can be detectable before fulfilment of the conventional threshold.

### 6.2. Antithrombin

Antithrombin has a strong mechanistic rationale but uneven evidence. It is synthesised predominantly by the liver and falls both through impaired synthesis and through consumption. Severe depression of antithrombin III in AFLP was described in 1983 in four women, in two of whom intravascular coagulation came under prompt control coincident with a rise in antithrombin III after plasma transfusion [68]. Minakami and colleagues proposed adding an antithrombin activity below 65% to the diagnostic criteria, arguing that its omission delays diagnosis until life-threatening complications appear [43], and a later pooled analysis of Japanese case reports found a median antithrombin activity of 23.0% (interquartile range 17.0–33.0) alongside a median fibrinogen of 82.0 mg/dL and an INR of 1.59 [42]. Low antithrombin was described as a distinguishing feature of AFLP, compared with HELLP syndrome, in a small comparative series [69] and has also been reported in a quaternary Australian series [73]; these observations describe an association, not diagnostic accuracy.

The contrary evidence is of two kinds, which should be kept distinct. As a prognostic marker within established AFLP, antithrombin III had an AUC of 0.420 (95% CI 0.294–0.546; p = 0.202) for a composite adverse outcome in 92 Chinese women, a confidence interval that spans 0.5 and therefore indicates no demonstrable discrimination, against 0.837 (95% CI 0.751–0.924; *p* < 0.001) for INR; the composite itself included coagulopathy and disseminated intravascular coagulation [4]. As a diagnostic marker, the evidence remains anecdotal: a biopsy-proven case had an antithrombin III of 87% with a fibrinogen below 50 mg/dL [23], and the 65% threshold was proposed rather than derived from a formal diagnostic-accuracy analysis and has not been validated. Antithrombin is therefore a biologically plausible candidate marker of hepatic synthetic failure whose timing and diagnostic operating characteristics remain undetermined and whose prognostic value was not supported in the one adequately sized cohort that examined it.

### 6.3. Conjugated Hyperbilirubinaemia

Jaundice is the feature clinicians recognise, but its diagnostic content probably lies in the conjugated fraction. Direct bilirubin constituted approximately 85% of total bilirubin in 133 women [5] and approximately 62% in 106 [38]. Total bilirubin was 4.4 versus 0.9 mg/dL in AFLP compared with HELLP syndrome [40], and total bilirubin is among the four analytes proposed for differentiating the two conditions on admission, alongside fibrinogen, creatinine and cholesterol [67]. Within AFLP, a prognostic rather than diagnostic setting, total bilirubin predicted progression to acute liver failure with an AUC of 0.80 (95% CI 0.72–0.88) at a cut-off of 61.58 µmol/L [5]. The Swansea threshold of 14 µmol/L sits close to the upper limit of normal and carries little specificity; a conjugated-predominant pattern with a substantially higher threshold might be more informative, but no such threshold has been derived for early diagnosis.

### 6.4. Hypoglycaemia

Hypoglycaemia is potentially one of the more specific routinely available abnormalities because failure of hepatic gluconeogenesis has few alternative explanations in an otherwise well third-trimester woman, although its specificity has not been estimated directly. Glucose was 85.0 versus 98.5 mg/dL in AFLP versus HELLP syndrome (*p* < 0.001) [40]. Glucose dysregulation is common once the illness is established: abnormal glucose was recorded in 89 of 133 women (66.9%), with hypoglycaemia in 76 (57.1%) and hyperglycaemia in 13 (9.8%) [14]. Its behaviour earlier in the illness appears quite different, and this deserves emphasis because it cuts against the intuition that hypoglycaemia is an early warning sign. In a separate case–control sample, approximately three days before the final Swansea-based diagnosis, glucose below 72 mg/dL was present in only 5 of 41 women with AFLP, against 3 of 172 controls (12.2% versus 1.7%, reported as 2.3%; *p* < 0.05) [34]. It was statistically discriminating but of little value as a screen, and adding it to the diagnostic model reduced sensitivity from 97.6% to 12.2% [34]. Mean glucose in a cohort of 106 was 4.4 ± 2.0 mmol/L, within the reference interval, although the timing of sampling was not specified [38]. Hypoglycaemia may therefore support suspicion of AFLP when present, but its absence cannot exclude the disease; the available retrospective data do not establish its timing of onset.

### 6.5. Lipids

Hypocholesterolaemia is a preliminary candidate marker of the same synthetic failure that lowers the coagulation factors, and it has the useful property of moving in the opposite direction to normal pregnancy, in which cholesterol rises. Low cholesterol and low triglycerides were described as distinguishing features of AFLP from HELLP syndrome in an early comparative series [69]. Cholesterol is also one of the four admission analytes proposed, on the basis of clinical observation, in a review by the Parkland group [67]. A recent case report of presumed AFLP argues that hypocholesterolaemia may differentiate AFLP from HELLP syndrome and be particularly valuable in resource-limited settings [70]. A single-centre study reporting lower low-density lipoprotein cholesterol in ‘AFLP’ than in controls cannot be interpreted, for the reasons discussed in Section 6.7, and the difference was not reproduced in its own test cohort [71]. The evidence base remains thin—largely single centres, small numbers and inconsistent timing of sampling—so its early clinical usefulness has not been established.

### 6.6. Angiogenic Markers

An sFlt-1 cut-off of 31,100 pg/mL separated AFLP from HELLP syndrome with 100% sensitivity and specificity in the Swiss series [36]. That figure should be read with caution: it was derived in six women with AFLP, it is an optimally chosen threshold in the sample that generated it and has not been externally validated, and the authors themselves state that corroboration is required. An independent Dutch group replicated markedly elevated concentrations [65,66], which supports the direction of the association but does not validate the cut-off. The practical obstacles are availability and turnaround. The conceptual attraction is that sFlt-1 is not a measure of hepatic failure, and is therefore partly independent of the synthetic axis—which is what a complementary criterion should be.

### 6.7. Imaging

Imaging deserves less weight than its position among the criteria implies. In the classical radiological series, findings consistent with hepatic fatty infiltration were present in 3 of 11 patients examined by ultrasound, 5 of 10 by computed tomography and none of 5 by magnetic resonance imaging. The authors concluded that detection of hepatic fat was limited and did not contribute to management in their series [41]. Modern practice has not improved on this: only 20 of 133 women (15.0%) had ultrasonographic evidence of fatty change, with four more showing slight parenchymal enhancement on computed tomography [5]. Newer techniques do not obviously rescue the situation: in a biopsy-proven case, the controlled attenuation parameter was 196 dB/m, corresponding to steatosis grade S0, even though ultrasonography and computed tomography suggested fatty infiltration; the authors suggest that attenuation-based methods may under-detect microvesicular fat [23]. Doppler assessment has been proposed as an alternative: one study reported reduced portal venous velocity (22.9 versus 27.7 cm/s) and a nomogram with an AUC of 0.88 in derivation and 0.86 in an internal test set [71]. Its ‘AFLP’ group, however, was drawn from healthy antenatal attendees, had normal aminotransferases and bilirubin and was not defined by the Swansea or any other stated criteria, so the finding cannot yet be regarded as evidence about AFLP. In practice, the imaging criterion is probably satisfied more often by ascites, a typically later feature, than by ultrasonographic steatosis.

### 6.8. Multivariable Models Built from Routine Clinical Data

We identified only one study that has constructed and reported a diagnostic model from variables measured before the Swansea threshold was reached (Section 4.1) [34]. It has not been externally validated, no AUC or calibration was reported, and its control group was assembled by diagnosis rather than by presenting syndrome. The authors acknowledged that a three-day lead was short. Most of the other models we identified are prognostic rather than diagnostic and should not be confused with it. The Model for End-stage Liver Disease predicted maternal mortality with an AUC of 0.948 in one cohort [38]. A five-variable index measured 72 h after delivery predicted three-month mortality with an AUC of 0.874, against 0.781 for the Model for End-stage Liver Disease [74]. Table 3 summarises the certainty of the principal statements across the domains reviewed here, using the categories defined in Section Approach to Literature Identification and Certainty Assessment.

**Table 3:** Summary of the evidence reviewed, with a qualitative judgement of the certainty of each statement.

Statement	Certainty ^a^	Principal Basis
The Swansea criteria are frequently fulfilled once AFLP is clinically established	Probable	Diagnostic accuracy: 100% sensitivity against biopsy in a small, severe-spectrum validation series (17 cases). Criterion fulfilment: 90.4% of 73 clinically diagnosed women on retrospective rescoring and 13 of 14 s-trimester cases; because the criteria also define the disease in most cohorts, fulfilment rates are not unbiased sensitivity estimates [13,22,33]
Sensitivity against an independent reference standard across the disease spectrum, including early disease	Knowledge gap	Only one small, severe-spectrum biopsy series identified; prospective validation at initial presentation remains a key evidence gap [22,34]
Predictive values reported for the criteria are prevalence-dependent and cannot be directly transported to a lower-prevalence triage population without recalibration	Established	Arithmetic property of predictive values; the reported counts correspond to a prevalence of approximately 71% in the validation sample, against approximately 3.5% among women with abnormal liver tests in pregnancy in one prospective cohort [3,22]
Specificity against HELLP syndrome and viral acute liver failure appears limited	Probable	Diagnostic accuracy: specificity 57% in the biopsy series (4 of 7 non-cases classified correctly). Criterion fulfilment in other conditions: a direct comparative study concluding that HELLP syndrome and viral acute liver failure also meet the criteria, and a multicentre acute liver failure registry that included no cases of hepatitis A–E, in which the criteria were met by every woman with pregnancy-associated liver disease and by virtually all women with other causes. Not estimated in an undifferentiated population [22,25,32]
The six-criterion threshold may remain unmet earlier in the illness, when biochemical abnormalities are already detectable, and be met more often once disease is clinically established (conceptual interpretation)	Emerging	One retrospective case–control study (*n* = 41): no woman met the threshold approximately three days before the Swansea-based final diagnosis, not necessarily at initial presentation, which is partly expected by design, whereas coagulation, aminotransferase and bilirubin abnormalities were already present; 90.4% of a separate cohort met the threshold on retrospective rescoring; consistent with the item composition, but within-patient progression has not been demonstrated [13,34]
Criterion accumulation may carry prognostic information, with higher criterion counts appearing to track disease severity	Emerging	AUCs of 0.69–0.89 for maternal and perinatal complications and treatment requirements at in-sample optimal cut-offs of 7–10 in a single retrospective cohort, not independently replicated; guideline listing of >7 criteria among predictors of intensive care admission; strong correlation with sFlt-1 in six women; not developed or validated as a severity instrument [12,13,36]
Coagulopathy disproportionate to the platelet count favours AFLP over HELLP syndrome	Probable	Approximately half of women with AFLP are not thrombocytopenic at presentation; consistent direction across comparative cohorts and a pooled case-report analysis [40,42,43]
Conventional imaging has shown limited ability to detect hepatic fatty changes in the available AFLP series	Established	Consistent across the classical radiological series and a contemporary cohort, and mechanistically expected for microvesicular fat [5,41]; attenuation-based quantification was falsely reassuring in a biopsy-proven case [23]
Foetal fatty acid oxidation defects were documented in a minority of selected AFLP pregnancies tested; conversely, maternal liver disease was absent in most pregnancies carrying an affected foetus	Established	Confirmed defect in 35.1% of 77 tested pregnancies, a selected sample rather than a population prevalence; 84% of pregnancies carrying an affected foetus have no maternal liver disease [48,59]
A maternal metabolite-level signature of fatty acid oxidation failure could precede some conventional laboratory abnormalities	Knowledge gap (hypothesis)	Biologically plausible; increased maternal long-chain and 3-hydroxy fatty acids described in established AFLP, but timing and diagnostic performance not evaluated (Section 5.3) [62,63]
Antithrombin activity below 65% is a valid early diagnostic criterion	Knowledge gap	Proposed but neither derived from a diagnostic-accuracy analysis nor validated; the only cohort-level AUC (0.420, *p* = 0.202, *n* = 92) concerns prognosis within AFLP and does not address diagnosis [4,43]
Hypocholesterolaemia may help differentiate AFLP from HELLP syndrome	Emerging	Consistent direction in a small comparative series, a single case report and an expert review proposing admission discriminators; no derived threshold and no prospective evaluation [67,69,70]
sFlt-1 is markedly higher in AFLP than in HELLP syndrome	Association (direction): Probable; threshold: Emerging	Two independent small cohorts with a concordant, unexpected direction of effect; the threshold was derived in six AFLP cases and has not been externally validated [36,66]
Earlier diagnosis improves maternal survival	Knowledge gap	Observational associations only, vulnerable to reverse causation and confounding by severity; no experimental evidence identified [6,14,44]
The distributions of ammonia and lactate in AFLP	Knowledge gap	Rarely reported in modern cohorts; ammonia did not differ from HELLP syndrome in one comparative study; the published lactate threshold carries an unresolved unit discrepancy [12,35,40]
Recurrence in subsequent pregnancy is uncommon	Probable	One recurrence in 35 subsequent pregnancies across ten series, and none in nine subsequent pregnancies to seven women in a further series, against five of 24 women in a patient survey [49,73]

^a^ Qualitative categories assigned by the authors using the criteria described in Section Approach to Literature Identification and Certainty Assessment, not formal GRADE ratings: Established, a well-supported finding with reasonably consistent independent evidence, or a mathematical relationship, applying to the specific statement rather than automatically to early diagnostic usefulness; Probable, credible clinical evidence with important limitations in design, precision, replication or applicability; Emerging, preliminary clinical findings requiring independent confirmation; Knowledge gap, insufficient direct evidence to answer the stated question reliably. AFLP, acute fatty liver of pregnancy; AUC, area under the receiver operating characteristic curve; GRADE, Grading of Recommendations Assessment, Development and Evaluation; HELLP, haemolysis, elevated liver enzymes and low platelets; sFlt-1, soluble fms-like tyrosine kinase-1.

## 7. Towards Stage-Specific Criteria: A Research Perspective

### 7.1. Why a Single Instrument Is Unlikely to Serve Both Purposes Well

The Swansea criteria appear to perform well at what they were built to do, which is to classify AFLP consistently once the illness is expressed. They are less well suited to a task they were not designed for: flagging the disease before overt hepatic and multiorgan failure has developed. The two tasks have different requirements. A case definition should be specific, stable over time and comparable across studies; an early-recognition rule should be sensitive, computable from rapidly available tests and tolerant of false positives that serial testing could resolve. Counting the same fourteen items for both purposes makes it unlikely that either is optimised.

The arithmetic of low prevalence illustrates the point. In illustrative Bayes calculations (Section 3.1; Supplementary Table S6), assuming an AFLP prevalence of approximately 3.5% among women with abnormal liver tests in pregnancy [3] and unchanged sensitivity and specificity across populations, the Swansea criteria would have a PPV of approximately 8%, whereas the earlier-timepoint model of Zhong and colleagues would have one of approximately 55% [22,34]. Neither figure is an observed PPV in a contemporary clinical population, and the transport assumption is questionable for spectra as different as a biopsy series and a triage population. The qualitative message nonetheless survives: specificity, not sensitivity, is the binding constraint, and a criteria set with a specificity near 57% is unlikely to function as a stand-alone diagnostic rule in an undifferentiated population.

### 7.2. Proposed Design Principles

We propose eight design principles for future early-disease criteria, derived from the evidence reviewed above.

Define the target population explicitly, since diagnostic performance cannot be interpreted without it. A defensible frame is any woman from ≥20 weeks of gestation through the early postpartum period with new hepatic dysfunction or a compatible constitutional prodrome; the precise postpartum limit would need to be prespecified in any validation study. Both bounds are informed by the literature: AFLP diagnosed between 20 and 27 weeks and 6 days is documented, with 13 of 14 such women meeting the Swansea criteria [33], and a substantial minority of cases are recognised only after delivery [14,19,44].

Anchor early recognition on hepatic synthetic function. This is where the mechanism suggests early quantitative change, and where pregnancy physiology works in the clinician’s favour, since a procoagulant baseline makes any prolongation of clotting times conspicuous [40,42].

Use pregnancy-specific and laboratory-specific reference intervals. This is an explicit recommendation of a study that specifically examined the Swansea thresholds against normal pregnancy physiology [20], and trimester-specific intervals for the relevant analytes are available [75]. It changes the behaviour of at least the urate, leucocyte and creatinine items, and of fibrinogen, which is not a Swansea item but would enter any rule anchored on synthetic function.

Consider trajectory rather than a single crossing. A rule incorporating deterioration across consecutive measurements would use a characteristic property of the disease—its progression. Serial assessment may improve discrimination by incorporating disease trajectory, but this requires prospective validation.

Separate diagnosis from severity assessment. Encephalopathy, ascites and a creatinine above 150 µmol/L are predominantly features of advanced disease and could be assessed on a parallel severity axis, where the prognostic evidence places them [12,13,38]. Retaining them in an early recognition rule may delay its fulfilment.

Break the circularity. Histology should not be an item in a construct that is validated against histology. Where biopsy is obtained, it should serve as a reference standard only, as the Vellore investigators describe having done by applying the remaining thirteen items antenatally [21,22].

Weight the items. An unweighted count scores vomiting, thirst and a leucocytosis above a conjugated bilirubin of 90 µmol/L with an INR of 2.0 and a fibrinogen of 1.2 g/L, three points to two: each item scores a single point whatever its magnitude, and fibrinogen is not counted.

Keep a stable confirmatory definition. The existing criteria should be retained unchanged for surveillance and comparability with more than two decades of literature: the proposal is to add a stage, not to replace an instrument.

### 7.3. A Conceptual Framework for Stage-Specific Recognition

**Figure 3:**
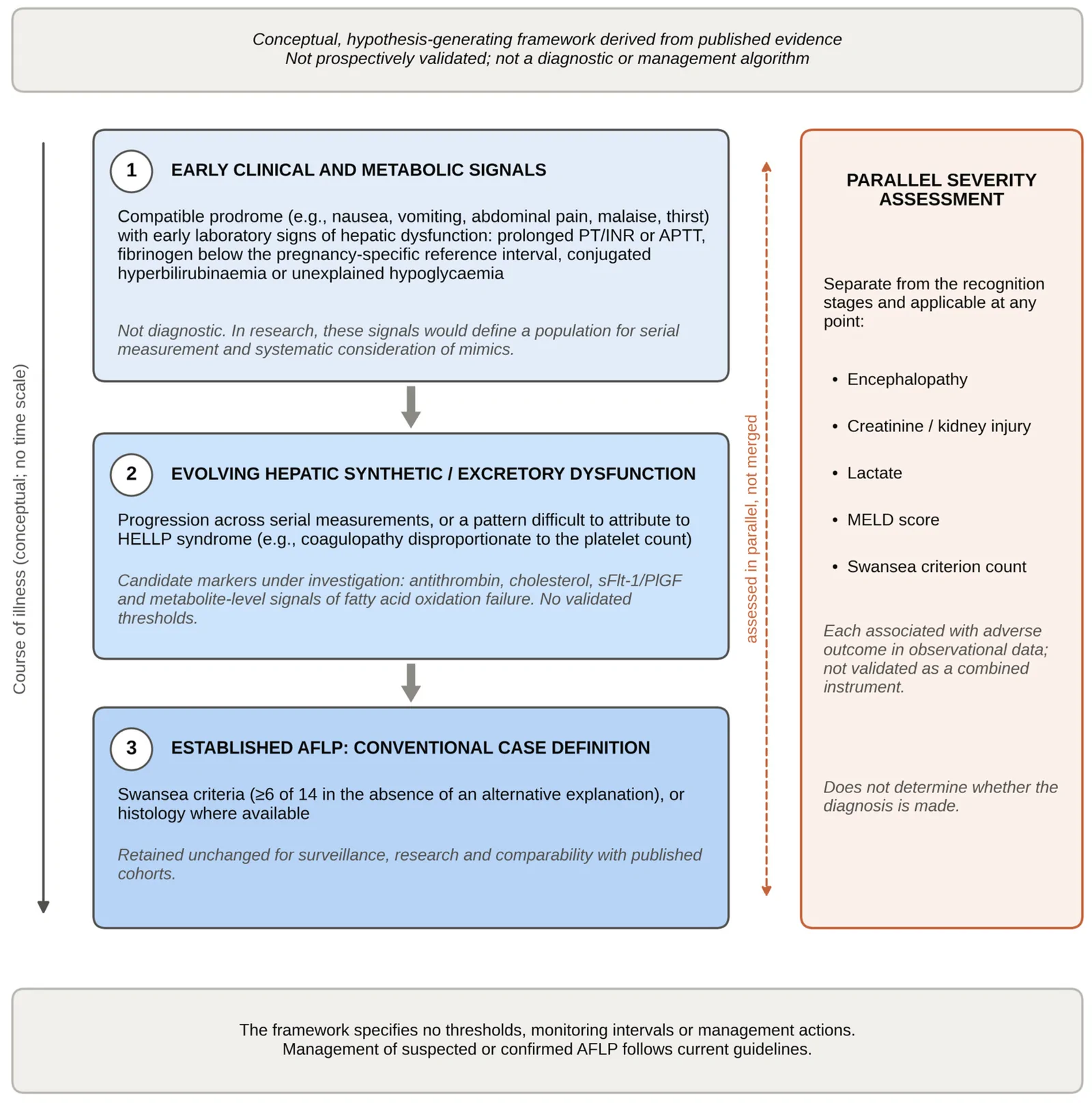
Conceptual framework for stage-specific recognition of acute fatty liver of pregnancy (AFLP), proposed for prospective research. Three stages are distinguished: early clinical and metabolic signals; evolving hepatic synthetic and excretory dysfunction; and established AFLP as defined by the conventional case definition (the Swansea criteria, or histology where available), which the framework retains and complements. Severity assessment is shown as a separate, parallel process that is not merged with the recognition stages. The candidate markers listed are those appraised in Table 2; none has a validated threshold for this purpose. Thirst and unexplained hypoglycaemia are listed in the first stage for inclusiveness, although their timing is insufficiently characterised (Table 1). The framework is hypothesis-generating and has not been prospectively validated; it does not replace the Swansea criteria and is not a validated recognition rule or a management algorithm. Management of suspected or confirmed AFLP should follow current guidelines [11,12,15]. Diagnostic and management pathways proposed by other authors differ in purpose and structure [21,34]. The figure was drawn de novo. APTT, activated partial thromboplastin time; HELLP, haemolysis, elevated liver enzymes and low platelets; INR, international normalised ratio; MELD, Model for End-stage Liver Disease; PlGF, placental growth factor; PT, prothrombin time; sFlt-1, soluble fms-like tyrosine kinase-1.

Figure 3 illustrates how these principles might combine into three stages of recognition alongside a parallel severity assessment. The framework is conceptual, hypothesis-generating and intended for prospective research; it complements the retained conventional case definition, does not replace the Swansea criteria and is not a validated recognition rule or a management algorithm.

The first stage, early clinical and metabolic signals, concerns a woman within the defined population who has a compatible constitutional prodrome together with laboratory evidence of hepatic dysfunction, such as prolongation of the prothrombin time, INR or activated partial thromboplastin time, a fibrinogen below the pregnancy-specific reference interval [75], conjugated hyperbilirubinaemia or unexplained hypoglycaemia, although the timing of hypoglycaemia remains insufficiently characterised and it was uncommon at the prediagnostic timepoint examined in one retrospective study (Section 6.4). These signals are not diagnostic. The stage is deliberately inclusive, and its role in a research setting would be to define a population for serial measurement and for systematic consideration of the mimics summarised in Supplementary Table S3.

The second stage, evolving hepatic synthetic and excretory dysfunction, is characterised by progression of these indices across serial measurements or by a pattern difficult to attribute to HELLP syndrome, of which the most informative is probably coagulopathy disproportionate to the platelet count, since around half of women with AFLP are not thrombocytopenic at presentation [42,43]. Candidate additional markers, none validated for this purpose, include antithrombin activity [43], cholesterol [67,69,70], sFlt-1 [36,66] and metabolite-level signals of fatty acid oxidation failure (Section 5.3).

The third stage, established AFLP, remains defined by the conventional case definition—the Swansea criteria, or histology where available.

Severity assessment runs in parallel with, and separately from, the recognition stages: encephalopathy, lactate, creatinine, the Model for End-stage Liver Disease score and the Swansea criterion count itself have each been associated with adverse outcome [12,13,35,38]. The framework does not specify thresholds, monitoring intervals or management actions; the management of suspected or confirmed AFLP, including the timing of delivery, should follow current guidelines [11,12,15].

The purpose of the framework is to make the underlying claim testable: that a stage-specific rule anchored on hepatic synthetic function could identify AFLP earlier than the Swansea threshold without an unacceptable loss of specificity in a realistically defined triage population.

## 8. Discussion

### 8.1. Principal Contribution

The principal contribution of this synthesis is to treat a familiar instrument as a potentially time-dependent construct and to argue from published data that its diagnostic and prognostic functions are not separated as currently used. Four elements support this interpretation: the two-by-two table of the biopsy-anchored validation, which places its widely quoted predictive values at a prevalence roughly twenty-fold higher than that observed among women with abnormal liver tests in pregnancy in one prospective cohort [3]; the coagulation and antithrombin literature from obstetric medicine, which identifies a preserved platelet count, not included in the criteria, as a feature favouring AFLP over HELLP syndrome; a qualitative mapping of each criterion onto its mechanism and interpreted timing, which offers a possible structural explanation for the hypothesised delay in fulfilment; and a reading of the criteria through failed mitochondrial β-oxidation, which identifies metabolite-level signals that have yet to be tested as early discriminators.

### 8.2. Limitations

The limitations are substantial and mostly reside in the underlying evidence. AFLP is rare, so the entire diagnostic literature rests on small samples: 24 analysed patients in the biopsy-anchored validation [22], 41 in the pre-threshold case–control study [34], and six in the sFlt-1 derivation series [36]. The biopsy validation is a two-page correspondence in which only seven patients lacked steatosis, and its operating characteristics have not been independently reproduced. Almost all cohorts are retrospective and single-centre, and almost all define AFLP by the very criteria under examination, an incorporation bias that makes independent estimation of sensitivity or specificity impossible; most comparative samples were also of severe spectrum or selected by diagnosis, a source of spectrum bias. Laboratory methods, reference intervals and units differ across studies and across four decades. Populations differ in ways that matter: the balance between AFLP and viral acute liver failure is entirely different in South Asia and in northern Europe [27,76,77], so transportability of any rule cannot be assumed. Publication and citation bias plausibly favour reports in which the criteria performed well or in which a proposed marker discriminated. The timing assignments in Table 1 and Figure 1 are qualitative interpretations rather than empirically measured onset times, and the certainty categories in Table 2 and Table 3 are qualitative judgements rather than formal evidence grades. The absence of a formal study-level risk-of-bias assessment limits the reproducibility and interpretability of these qualitative assignments and the strength of the evidence hierarchy in Table 2 and Table 3.

The narrative design imposes its own constraint. Selection of sources reflects the judgement of the authors, the iterative identification process cannot be reproduced exactly, and a differently conducted synthesis might weight the same literature differently. We have tried to counter this by seeking out and reporting the evidence that contradicts the central argument: the prognostic antithrombin AUC of 0.420 [4]; the 90.4% criterion fulfilment on retrospective rescoring [13]; and the absence, to our knowledge, of any experimental evidence that earlier diagnosis improves survival.

## 9. Future Directions

The most consequential gap is a study design, not a marker. What is needed is a prospective, multicentre cohort enrolling consecutive women in the population defined above, irrespective of the suspected cause, with a prespecified panel measured at first presentation and at fixed intervals thereafter, and the final diagnosis adjudicated blind to those measurements against an a priori composite standard. Such a study would permit estimation of the specificity of the Swansea criteria in the population that uses them, and would allow time-to-threshold to be treated as an outcome in its own right. At an incidence of 5 per 100,000 maternities [1], an international collaboration built on existing obstetric surveillance infrastructure would probably be needed to recruit adequately.

Four narrower priorities follow. Antithrombin requires a formal diagnostic-accuracy study in this population, with a derived rather than assumed threshold and reported confidence intervals [43]. Ammonia and lactate should simply be measured and reported in prospective cohorts, since one is a diagnostic criterion and the other a guideline-endorsed severity marker [12], yet the distribution of neither is well described in AFLP. The sFlt-1 findings need replication in a cohort large enough to derive a threshold independently of the six-case sample that generated it [36,66]. And, the metabolite-level signals outlined in Section 5.3 should be measured at presentation within the same prospective cohorts, so that targeted or untargeted metabolomic discrimination is tested against an adjudicated reference standard rather than against the Swansea criteria [62,63].

Finally, the long-term consequences of AFLP for mother and child remain under-characterised. Follow-up of 25 women over a mean of 54.5 months found no cirrhosis, and normalisation of liver and renal function [78]. Recurrence appears uncommon: one recurrence in 35 subsequent pregnancies across ten small series, set against five of 24 women in a survey [49]. That discrepancy—probably reflecting the difference between clinician-ascertained and patient-reported recurrence—is itself an unresolved question with direct implications for counselling.

## 10. Conclusions

The Swansea criteria are frequently fulfilled once AFLP is clinically established, but their accuracy against an independent reference standard is incompletely characterised, particularly during the earliest phase of disease. The evidence reviewed here can be grouped according to its strength (Table 3).

Established. Published predictive values reflect a prevalence of approximately 71% in the biopsy-anchored validation and cannot be directly transported to a lower-prevalence population without recalibration. Conventional imaging has shown limited ability to detect hepatic fatty changes in the available AFLP series. Foetal fatty acid oxidation defects have been confirmed in a minority of selected pregnancies investigated and do not account for all cases of maternal AFLP; their principal established clinical implications concern neonatal evaluation rather than maternal diagnosis.

Probable. Criterion fulfilment in HELLP syndrome, viral acute liver failure and other mimicking conditions raises concerns about the discriminatory specificity of the Swansea criteria. Coagulopathy disproportionate to the platelet count favours AFLP over HELLP syndrome, and a preserved platelet count should not be taken as evidence against AFLP. sFlt-1 concentrations appear to be higher in AFLP than in HELLP syndrome.

Emerging. On limited retrospective evidence, biochemical abnormalities, particularly of coagulation, appear to be detectable before the conventional threshold is fulfilled, and criterion accumulation may carry prognostic information. Hypocholesterolaemia, bile acids and an sFlt-1 threshold derived in six cases are preliminary candidate markers, none of which has been prospectively validated.

Knowledge gap. It remains unknown whether earlier diagnosis improves maternal survival, what diagnostic value antithrombin has, how ammonia and lactate are distributed in AFLP, and whether a maternal metabolite-level signature of fatty acid oxidation failure precedes conventional abnormalities. The framework proposed here is a hypothesis-generating construct for prospective research that complements, rather than replaces, the Swansea case definition. The Swansea criteria still require prospective evaluation at initial presentation in the population in which they are intended to be used.

## Figures and Tables

**Table 1:** Item-by-item appraisal of the Swansea criteria: domain, interpreted timing (the phase of the illness at which each item may become satisfiable), behaviour against normal pregnancy physiology, and contribution to discrimination from HELLP syndrome and pre-eclampsia.

#	Criterion (Threshold as Commonly Tabulated) ^a^	Domain	Interpreted Timing ^b^	Behaviour in Normal Pregnancy and Interpretive Caveat	Contribution to Discrimination from HELLP Syndrome or Pre-Eclampsia ^b^
1	Vomiting	Symptom	Potentially earlier	Common in normal pregnancy; the abnormal form is persistence beyond 20 weeks	Reported more often in AFLP than in HELLP syndrome, but not discriminating in isolation [7,10]
2	Abdominal pain	Symptom	Potentially earlier	Overlaps with labour, abruption and HELLP syndrome	Limited discriminatory value [7]
3	Polydipsia or polyuria	Symptom	Timing insufficiently characterised	Unusual complaint; may reflect transient diabetes insipidus	Potentially informative when present, given a short differential, but reported with widely varying frequency (polydipsia in 9 of 12 episodes in one classical series; polydipsia or polyuria in 9.4% of a modern cohort) [37,38,39]
4	Encephalopathy	Systemic organ failure	Predominantly later (advanced disease)	Absent until hepatic failure is advanced	Of little value for early recognition [34]
5	Bilirubin >14 µmol/L	Hepatic excretory	Potentially earlier	Threshold lies close to the upper limit of normal; the conjugated fraction is not specified	Frequently met but weakly discriminating at this threshold; magnitude and conjugated predominance appear more informative (4.4 versus 0.9 mg/dL in AFLP versus HELLP syndrome) [5,40]
6	Hypoglycaemia <4 mmol/L	Hepatic metabolic	Timing insufficiently characterised	Few alternative explanations in an otherwise well third-trimester woman	Potentially useful for rule-in, but infrequent earlier in the illness: hypoglycaemia in 57.1% of one multicentre cohort once established, yet in only 12.2% of a separate cohort approximately three days before the final Swansea-based diagnosis [14,34,38]
7	Urate >340 µmol/L	Non-specific laboratory	Potentially earlier, often for reasons unrelated to AFLP	Rises physiologically through gestation and is a recognised feature of pre-eclampsia	Pulls towards the competing diagnosis; did not differ between AFLP and HELLP syndrome in the comparative study that reported it [20,40]
8	Leucocytosis >11 × 10^9^/L	Non-specific laboratory	Potentially earlier	Common in normal labour and in intercurrent infection	Limited discriminatory value [20]
9	Ascites or bright liver on ultrasound	Imaging	Ascites: predominantly later; bright liver: timing insufficiently characterised; detection limited in available series	Hepatic fat detected in 3 of 11 patients by ultrasound and 5 of 10 by computed tomography in the original radiological series and in 15% of a modern cohort; abnormal ultrasound in 4 of 41 women approximately three days before the final diagnosis; magnetic resonance imaging was positive in none of five [5,34,41]	The imaging item may be satisfied more often by ascites, a typically later feature, than by bright liver [5,41]
10	Aspartate or alanine aminotransferase >42 IU/L	Non-specific laboratory	Potentially earlier	Threshold lies far below the values actually observed in AFLP (typically 250–300 U/L)	Frequently met, with little discriminatory value at this threshold; the disproportion between cytolysis and synthetic failure appears more informative [5,40]
11	Ammonia >47 µmol/L	Hepatic metabolic	Timing insufficiently characterised (rarely measured)	Its distribution in AFLP is poorly described	Did not differ between AFLP and HELLP syndrome in the single comparative study that measured it [40]
12	Creatinine >150 µmol/L	Systemic organ failure	Predominantly later (advanced disease)	A marked elevation against the low creatinine of normal pregnancy	More frequent in AFLP (acute kidney injury in 55–75% versus 7–20% of pre-eclampsia or HELLP syndrome) [2]
13	Coagulopathy (prothrombin time >14 s or activated partial thromboplastin time >34 s)	Hepatic synthetic	Potentially earlier [34]	Pregnancy is a procoagulant state, so any prolongation is already abnormal	Potentially among the more discriminating items (prothrombin time 14.1 versus 9.5 s in AFLP versus HELLP syndrome); fibrinogen (193 versus 408.1 mg/dL) and antithrombin are not themselves counted [40,42,43]
14	Microvesicular steatosis on liver biopsy	Histology	Predominantly later (rarely available; usually postpartum, if at all)	Not performed at all in a modern series of 133 women; generally obtained postpartum when performed [5,23]	Serves as reference standard; counting it would make biopsy-anchored validation circular, which the Vellore validation avoided by applying the remaining thirteen items antenatally [21,22]

^a^ Six or more items are required in the absence of an alternative explanation. Published tabulations of the criteria differ in the units printed for the leucocyte, urate and ammonia items; the thresholds shown follow the International System of Units, with enzyme activity in IU/L, and comparative values are given as originally reported. ^b^ Interpreted timing and contribution to discrimination are qualitative interpretations of the cited sources and of the pathophysiology outlined in Section 5, not empirically measured onset times or formal accuracy estimates. ‘Potentially earlier’ denotes items that can be abnormal before the diagnostic threshold is met; ‘predominantly later’ denotes items mainly associated with advanced disease or, for histology, with postpartum availability; ‘timing insufficiently characterised’ denotes items for which the available data do not support a timing assignment. AFLP, acute fatty liver of pregnancy; HELLP, haemolysis, elevated liver enzymes and low platelets.

## Data Availability

No new data were created or analysed in this study. Data sharing is not applicable to this article.

## Supplementary Materials

The following supporting information can be downloaded at https://www.mdpi.com/article/10.3390/metabo16100799/s1. Table S1: Characteristics and principal findings of the cohorts, series and evidence syntheses informing this review; Table S2: Laboratory values in acute fatty liver of pregnancy organised by functional domain, with a single-centre comparison against HELLP syndrome; Table S3: Conditions in the second half of pregnancy that can fulfil Swansea-type features, and the investigations that discriminate them; Table S4: Evidence linking foetal fatty acid oxidation defects to maternal liver disease in pregnancy; Table S5: SANRA items and where each is addressed in the present narrative review (descriptive checklist); Table S6: Illustrative model-based calculations of predictive values across a range of disease prevalences.

## Abbreviations

The following abbreviations are used in this manuscript:

ACG	American College of Gastroenterology
ADAMTS13	a disintegrin and metalloproteinase with a thrombospondin type 1 motif, member 13
AFLP	acute fatty liver of pregnancy
ALF	acute liver failure
ALT	alanine aminotransferase
APTT	activated partial thromboplastin time
AST	aspartate aminotransferase
AUC	area under the receiver operating characteristic curve
CI	confidence interval
CPT-I	carnitine palmitoyltransferase I
FAO	fatty acid oxidation
FIGO	International Federation of Gynecology and Obstetrics
GRADE	Grading of Recommendations Assessment, Development and Evaluation
HELLP	haemolysis, elevated liver enzymes and low platelets
INR	international normalised ratio
IQR	interquartile range
LCHAD	long-chain 3-hydroxyacyl-coenzyme A dehydrogenase
LDL	low-density lipoprotein
MCAD	medium-chain acyl-coenzyme A dehydrogenase
MELD	Model for End-stage Liver Disease
MTP	mitochondrial trifunctional protein
NPV	negative predictive value
PlGF	placental growth factor
PPV	positive predictive value
PT	prothrombin time
RR	relative risk
SANRA	Scale for the Assessment of Narrative Review Articles
SCAD	short-chain acyl-coenzyme A dehydrogenase
sFlt-1	soluble fms-like tyrosine kinase-1
UKOSS	United Kingdom Obstetric Surveillance System

## References

[1] Knight M., Nelson-Piercy C., Kurinczuk J.J., Spark P., Brocklehurst P., on behalf of UKOSS (2008). A prospective national study of acute fatty liver of pregnancy in the UK. Gut.

[2] Rajput M., Bachani S., Suri J., Bharti R., Kumar A., Kumar R., Verma H., Prasad P. (2025). Acute kidney injury associated with acute fatty liver of pregnancy: An update on a rare clinical entity. World J. Nephrol..

[3] Ch’ng C.L., Morgan M., Hainsworth I., Kingham J.G.C. (2002). Prospective study of liver dysfunction in pregnancy in Southwest Wales. Gut.

[4] Zhai P., Hu J., Ou X., Zhou L., Pan X. (2026). A retrospective analysis of coagulation function indicators in acute fatty liver of pregnancy. Front. Med..

[5] Lan J., Feng W., Liao S., Luo X., Huang W., Huang Z., Jin S., Liu K., Chen D., Chen Y. (2025). Risk factors for acute fatty liver of pregnancy complicated by acute liver failure: A retrospective study of 133 cases. BMC Pregnancy Childbirth.

[6] Li L., Huang D., Xu J., Li M., Zhao J., Shi Q., Guo Q. (2023). The assessment in patients with acute fatty liver of pregnancy (AFLP) treated with plasma exchange: A cohort study of 298 patients. BMC Pregnancy Childbirth.

[7] Nelson D.B., Yost N.P., Cunningham F.G. (2013). Acute fatty liver of pregnancy: Clinical outcomes and expected duration of recovery. Am. J. Obstet. Gynecol..

[8] Nelson D.B., Byrne J.J., Cunningham F.G. (2021). Acute Fatty Liver of Pregnancy. Obstet. Gynecol..

[9] Liu J., Ghaziani T.T., Wolf J.L. (2017). Acute Fatty Liver Disease of Pregnancy: Updates in Pathogenesis, Diagnosis, and Management. Am. J. Gastroenterol..

[10] White A., Nelson D.B., Cunningham F.G. (2024). Acute Fatty Liver of Pregnancy. Reprod. Med..

[11] Tran T.T., Ahn J., Reau N.S. (2016). ACG Clinical Guideline: Liver Disease and Pregnancy. Am. J. Gastroenterol..

[12] Nana M., Medina V., Maxwell C., McCormick C., Taliani G., Beuers U., Money D., Jacobsson B., Kapur A., Beyuo T. (2025). FIGO guideline on liver disease and pregnancy. Int. J. Gynaecol. Obstet..

[13] Tan J., Hou F., Xiong H., Pu L., Xiang P., Li C. (2022). Swansea criteria score in acute fatty liver of pregnancy. Chin. Med. J. (Engl.).

[14] Gao Q., Qu X., Chen X., Zhang J., Liu F., Tian S., Wang C. (2018). Outcomes and risk factors of patients with acute fatty liver of pregnancy: A multicentre retrospective study. Singap. Med. J..

[15] European Association for the Study of the Liver (2023). EASL Clinical Practice Guidelines on the management of liver diseases in pregnancy. J. Hepatol..

[16] Sarkar M., Brady C.W., Fleckenstein J., Forde K.A., Khungar V., Molleston J.P., Afshar Y., Terrault N.A. (2021). Reproductive Health and Liver Disease: Practice Guidance by the American Association for the Study of Liver Diseases. Hepatology.

[17] Li P., Chen Y., Zhang W., Yang H., Maternal-Fetal Medicine Committee, Chinese Society of Obstetrics and Gynecology, Chinese Medical Association (2021). CSOG MFM Committee Guideline: Clinical Management Guidelines for Acute Fatty Liver of Pregnancy in China (2021). Matern. Fetal Med..

[18] Baethge C., Goldbeck-Wood S., Mertens S. (2019). SANRA-a scale for the quality assessment of narrative review articles. Res. Integr. Peer Rev..

[19] Westbrook R.H., Dusheiko G., Williamson C. (2016). Pregnancy and liver disease. J. Hepatol..

[20] Morton A., Laurie J. (2018). Physiological changes of pregnancy and the Swansea criteria in diagnosing acute fatty liver of pregnancy. Obstet. Med..

[21] Goel A., Ch’ng C.L., Eapen C.E., Balasubramanian K.A., Elias E. (2018). Acute Fatty Liver of Pregnancy: Better Understanding of Pathogenesis and Earlier Clinical Recognition Results in Improved Maternal Outcomes. EMJ Hepatol..

[22] Goel A., Ramakrishna B., Zachariah U., Ramachandran J., Eapen C.E., Kurian G., Chandy G. (2011). How accurate are the Swansea criteria to diagnose acute fatty liver of pregnancy in predicting hepatic microvesicular steatosis?. Gut.

[23] Nosaka T., Saito A., Akazawa Y., Takahashi K., Naito T., Ohtani M., Imamura Y., Kudo S., Orisaka M., Yoshida Y. (2026). Case Report: Elastography-negative acute fatty liver of pregnancy diagnosed by histopathology and electron microscopy. Front. Med..

[24] Horani O., Mohiuddin I., Ahmed Z., Iqbal A., Asif M.W., Hassan M. (2025). Liver Biopsy During Pregnancy: A Review. Cureus.

[25] Devarbhavi H., Venkatachala P.R., Devamsh G.N., Shalimar, Aashik Y.S., Patil M., Acharya S.K. (2023). Swansea criteria evaluation in acute fatty liver of pregnancy, hemolysis elevated liver enzyme and low platelet syndrome, pre-eclampsia, and viral acute liver failure in pregnancy. Int. J. Gynaecol. Obstet..

[26] Jin H., Zhao Y., Zhang X., Wang B., Liu P. (2016). Case-fatality risk of pregnant women with acute viral hepatitis type E: A systematic review and meta-analysis. Epidemiol. Infect..

[27] Patra S., Kumar A., Trivedi S.S., Puri M., Sarin S.K. (2007). Maternal and fetal outcomes in pregnant women with acute hepatitis E virus infection. Ann. Intern. Med..

[28] Magawa S., Tanaka H., Furuhashi F., Maki S., Nii M., Toriyabe K., Ikeda T. (2020). A literature review of herpes simplex virus hepatitis in pregnancy. J. Matern. Fetal Neonatal Med..

[29] Kumar V., Madeswaran C., Sunkesula V., Kundrapu S. (2026). Pregnancy-Associated Thrombotic Thrombocytopenic Purpura: Diagnostic Pitfalls, Therapeutic Strategies, and Emerging Paradigms. Biomedicines.

[30] Liu L., Cui Y., Zhou Q., Zhao H., Li X. (2021). Hemophagocytic lymphohistiocytosis during pregnancy: A review of the literature in epidemiology, pathogenesis, diagnosis and treatment. Orphanet J. Rare Dis..

[31] Kokita A., Kuroda H., Azumaguchi R., Suzuki S., Kazuma S. (2025). A Rare Case of Hemophagocytic Lymphohistiocytosis in Pregnancy with Clinical Features Resembling Acute Fatty Liver of Pregnancy. Cureus.

[32] Casey L.C., Fontana R.J., Aday A., Nelson D.B., Rule J.A., Gottfried M., Tran M., Lee W.M. (2020). Acute Liver Failure (ALF) in Pregnancy: How Much Is Pregnancy Related?. Hepatology.

[33] Bourque L., Mahone M. (2025). Acute fatty liver in pregnancy presenting in the second trimester: A systematic literature review. Obstet. Med..

[34] Zhong Y., Zhu F., Ding Y. (2020). Early diagnostic test for acute fatty liver of pregnancy: A retrospective case control study. BMC Pregnancy Childbirth.

[35] Westbrook R.H., Yeoman A.D., Joshi D., Heaton N.D., Quaglia A., O’Grady J.G., Auzinger G., Bernal W., Heneghan M.A., Wendon J.A. (2010). Outcomes of severe pregnancy-related liver disease: Refining the role of transplantation. Am. J. Transplant..

[36] Trottmann F., Raio L., Amylidi-Mohr S., Mosimann B., Jarquin Campos A., Messerli F.H., Risch L., Baumann M.U. (2021). Soluble fms-like tyrosine kinase 1 (sFlt-1): A novel biochemical marker for acute fatty liver of pregnancy. Acta Obstet. Gynecol. Scand..

[37] Reyes H., Sandoval L., Wainstein A., Ribalta J., Donoso S., Smok G., Rosenberg H., Meneses M. (1994). Acute fatty liver of pregnancy: A clinical study of 12 episodes in 11 patients. Gut.

[38] Meng Z., Fang W., Meng M., Zhang J., Wang Q., Qie G., Chen M., Wang C. (2021). Risk Factors for Maternal and Fetal Mortality in Acute Fatty Liver of Pregnancy and New Predictive Models. Front. Med..

[39] Kennedy S., Hall P.M., Seymour A.E., Hague W.M. (1994). Transient diabetes insipidus and acute fatty liver of pregnancy. Br. J. Obstet. Gynaecol..

[40] Ang S.X., Chen C.P., Sun F.J., Chen C.Y. (2021). Comparison of maternal and neonatal outcomes between acute fatty liver of pregnancy and hemolysis, elevated liver enzymes and low platelets syndrome: A retrospective cohort study. BMC Pregnancy Childbirth.

[41] Castro M.A., Ouzounian J.G., Colletti P.M., Shaw K.J., Stein S.M., Goodwin T.M. (1996). Radiologic studies in acute fatty liver of pregnancy. A review of the literature and 19 new cases. J. Reprod. Med..

[42] Suzuki H., Ohkuchi A., Horie K., Ogoyama M., Usui R., Takahashi H., Fujiwara H. (2025). Acute fatty liver of pregnancy may be associated with non-productive coagulopathy of coagulation and fibrinolytic factors as well as disseminated intravascular coagulation with mild or no thrombocytopenia: Review of case reports in Japan. J. Obstet. Gynaecol. Res..

[43] Minakami H., Morikawa M., Yamada T., Yamada T., Akaishi R., Nishida R. (2014). Differentiation of acute fatty liver of pregnancy from syndrome of hemolysis, elevated liver enzymes and low platelet counts. J. Obstet. Gynaecol. Res..

[44] Lau H.H., Chen Y.Y., Huang J.P., Chen C.Y., Su T.H., Chen C.P. (2010). Acute fatty liver of pregnancy in a Taiwanese tertiary care center: A retrospective review. Taiwan J. Obstet. Gynecol..

[45] Castro M.A., Fassett M.J., Reynolds T.B., Shaw K.J., Goodwin T.M. (1999). Reversible peripartum liver failure: A new perspective on the diagnosis, treatment, and cause of acute fatty liver of pregnancy, based on 28 consecutive cases. Am. J. Obstet. Gynecol..

[46] Kushner T., Tholey D., Dodge J., Saberi B., Schiano T., Terrault N. (2019). Outcomes of liver transplantation for acute fatty liver disease of pregnancy. Am. J. Transplant..

[47] Yu C.B., Chen J.J., Du W.B., Chen P., Huang J.R., Chen Y.M., Cao H.C., Li L.J. (2014). Effects of plasma exchange combined with continuous renal replacement therapy on acute fatty liver of pregnancy. Hepatobiliary Pancreat. Dis. Int..

[48] Varotsis D., Araji S., Horgan R., Powel J.E., McLaren R., Kirmse B., Makhamreh M.M., Al-Kouatly H.B. (2026). Acute Fatty Liver of Pregnancy and Fetal Fatty Acid Oxidation Disorders: A Systematic Review. O&G Open.

[49] Morton A., Phillips L., Nisbet J., He J.W. (2025). The risk of recurrence of acute fatty liver of pregnancy—A review of the current literature. Obstet. Med..

[50] Ahmed S.J., Mudassar S., Ali S., Faryal N., Hasan T., Samad S. (2026). Fetal and Neonatal Complications Associated with Acute Fatty Liver of Pregnancy. Cureus.

[51] Cheng N., Xiang T., Wu X., Li M., Xie Y., Zhang L. (2014). Acute fatty liver of pregnancy: A retrospective study of 32 cases in South China. J. Matern. Fetal Neonatal Med..

[52] Wirawan W., Pribadi A. (2025). Diagnostic Challenges in Acute Fatty Liver of Pregnancy: Fever as a Key Symptom in Case Series from Indonesia. Am. J. Case Rep..

[53] Cao W., Chen T., Jiang W., Geng Y., Xie B., Wang Q., Wang X. (2022). Timely identification and successful treatment of acute fatty liver of pregnancy without obvious clinical symptoms: Case reports. Medicine.

[54] Wilcken B., Leung K.C., Hammond J., Kamath R., Leonard J.V. (1993). Pregnancy and fetal long-chain 3-hydroxyacyl coenzyme A dehydrogenase deficiency. Lancet.

[55] Treem W.R., Rinaldo P., Hale D.E., Stanley C.A., Millington D.S., Hyams J.S., Jackson S., Turnbull D.M. (1994). Acute fatty liver of pregnancy and long-chain 3-hydroxyacyl-coenzyme A dehydrogenase deficiency. Hepatology.

[56] Sims H.F., Brackett J.C., Powell C.K., Treem W.R., Hale D.E., Bennett M.J., Gibson B., Shapiro S., Strauss A.W. (1995). The molecular basis of pediatric long chain 3-hydroxyacyl-CoA dehydrogenase deficiency associated with maternal acute fatty liver of pregnancy. Proc. Natl. Acad. Sci. USA.

[57] Ibdah J.A., Bennett M.J., Rinaldo P., Zhao Y., Gibson B., Sims H.F., Strauss A.W. (1999). A fetal fatty-acid oxidation disorder as a cause of liver disease in pregnant women. N. Engl. J. Med..

[58] Yang Z., Yamada J., Zhao Y., Strauss A.W., Ibdah J.A. (2002). Prospective screening for pediatric mitochondrial trifunctional protein defects in pregnancies complicated by liver disease. JAMA.

[59] Browning M.F., Levy H.L., Wilkins-Haug L.E., Larson C., Shih V.E. (2006). Fetal fatty acid oxidation defects and maternal liver disease in pregnancy. Obstet. Gynecol..

[60] den Boer M.E.J., IJlst L., Wijburg F.A., Oostheim W., van Werkhoven M.A., van Pampus M.G., Heymans H.S.A., Wanders R.J.A. (2000). Heterozygosity for the common LCHAD mutation (1528G>C) is not a major cause of HELLP syndrome and the prevalence of the mutation in the Dutch population is low. Pediatr. Res..

[61] Mütze S., Ahillen I., Rudnik-Schoeneborn S., Eggermann T., Leeners B., Neumaier-Wagner P., Kuse S., Rath W., Zerres K. (2007). Neither maternal nor fetal mutation (E474Q) in the alpha-subunit of the trifunctional protein is frequent in pregnancies complicated by HELLP syndrome. J. Perinat. Med..

[62] Natarajan S.K., Ibdah J.A. (2018). Role of 3-Hydroxy Fatty Acid-Induced Hepatic Lipotoxicity in Acute Fatty Liver of Pregnancy. Int. J. Mol. Sci..

[63] Ramanathan R., Ibdah J.A. (2022). Mitochondrial Dysfunction and Acute Fatty Liver of Pregnancy. Int. J. Mol. Sci..

[64] Merritt J.L., Norris M., Kanungo S. (2018). Fatty acid oxidation disorders. Ann. Transl. Med..

[65] Neuman R.I., Hesselink E.R.M., Saleh L., van den Meiracker A.H., Danser A.H.J., Visser W. (2020). Angiogenic markers are elevated in women with acute fatty liver of pregnancy. Ultrasound Obstet. Gynecol..

[66] Neuman R.I., Saleh L., Verdonk K., van den Meiracker A.H., Russcher H., Metselaar H.J., Visser W., Danser A.H.J. (2021). Accurate Prediction of Total PlGF (Placental Growth Factor) from Free PlGF and sFlt-1 (Soluble Fms-Like Tyrosine Kinase-1): Evidence for Markedly Elevated PlGF Levels in Women with Acute Fatty Liver of Pregnancy. Hypertension.

[67] Byrne J.J., Seasely A.R., Nelson D.B., McIntire D.D., Cunningham F.G. (2022). Comparing acute fatty liver of pregnancy from hemolysis, elevated liver enzymes, and low platelets syndrome. J. Matern. Fetal Neonatal Med..

[68] Liebman H.A., McGehee W.G., Patch M.J., Feinstein D.I. (1983). Severe depression of antithrombin III associated with disseminated intravascular coagulation in women with fatty liver of pregnancy. Ann. Intern. Med..

[69] Vigil-De Gracia P. (2001). Acute fatty liver and HELLP syndrome: Two distinct pregnancy disorders. Int. J. Gynaecol. Obstet..

[70] Nik Ghazali N.N.I., Pavey J., Morton A. (2026). Hypocholesterolaemia as an early feature of presumed acute fatty liver of pregnancy. Obstet. Med..

[71] Zhao Y., Zhang E., Li D.H., Zhou Y.J., Jiang H., Jia X.C., Qin C. (2024). Reduced portal vein blood flow velocity in acute fatty liver of pregnancy. Front. Med..

[72] Nelson D.B., Yost N.P., Cunningham F.G. (2014). Hemostatic dysfunction with acute fatty liver of pregnancy. Obstet. Gynecol..

[73] Lamprecht A., Morton A., Laurie J., Lee W. (2018). Acute fatty liver of pregnancy and concomitant medical conditions: A review of cases at a quaternary obstetric hospital. Obstet. Med..

[74] Peng Q., Zhu T., Huang J., Liu Y., Huang J., Zhang W. (2024). Factors and a model to predict three-month mortality in patients with acute fatty liver of pregnancy from two medical centers. BMC Pregnancy Childbirth.

[75] Abbassi-Ghanavati M., Greer L.G., Cunningham F.G. (2009). Pregnancy and laboratory studies: A reference table for clinicians. Obstet. Gynecol..

[76] Devarbhavi H., Kremers W.K., Dierkhising R., Padmanabhan L. (2008). Pregnancy-associated acute liver disease and acute viral hepatitis: Differentiation, course and outcome. J. Hepatol..

[77] Alexander V., Benjamin S.J., Subramani K., Sathyendra S., Goel A. (2024). Acute liver failure in pregnancy. Indian J. Gastroenterol..

[78] Xiong H.F., Liu J.Y., Guo L.M., Li X.W. (2015). Acute fatty liver of pregnancy: Over six months follow-up study of twenty-five patients. World J. Gastroenterol..

