# Supplementary material for: Metabolic and Synthetic Dysfunction in Acute Fatty Liver of Pregnancy: Reappraising the Swansea Criteria—A Narrative Review
Source: Metabolites. 2026 Oct 21;16(10):799. doi: 10.3390/metabo16100799 (PMC13707121; doi:10.3390/metabo16100799)
Supplement: Supplementary file 1 [file metabolites-16-00799-s001.zip › metabolites-4629379-supplementary.pdf]

*Supplementary Materials*

# Metabolic and Synthetic Dysfunction in Acute Fatty Liver of Pregnancy: Reappraising the Swansea Criteria—A Narrative Review

Reference numbers in square brackets correspond to the reference list of the main manuscript.

**Table S1.** Characteristics and principal findings of the cohorts, series and evidence syntheses informing this review

| Study | Country / setting | Period | Design and size | Diagnostic basis | Principal quantitative findings |
|---|---|---|---|---|---|
| Ch'ng et al., 2002 [3] | United Kingdom, south-west Wales | 15 months | Prospective study of liver dysfunction across 4377 deliveries | Criteria devised in this study | Abnormal liver tests in 142 women (3% of deliveries); pre-eclampsia present in 68 but the only identifiable cause in 15; HELLP syndrome 30 (the commonest diagnosis; 29 with pre-eclampsia), obstetric cholestasis 23, sepsis 17, hyperemesis 11, AFLP 5 (approximately 1 in 875 deliveries); no maternal deaths, two intrauterine deaths; diagnostic categories as given in the authors' published correction |
| Reyes et al., 1994 [37] | Chile, Santiago | 18 years | Retrospective clinical series, 12 episodes in 11 women | Clinical features with liver biopsy in 11 episodes | Prevalence 1 per 15,900 deliveries; onset weeks 31–38; polydipsia in 9 episodes, pruritus in 7; positive intracellular fat staining in the four samples analysed, of 11 episodes biopsied; megamitochondria with paracrystalline inclusions in 4 samples; maternal mortality 0%, fetal mortality 58.3% |
| Knight et al., 2008 [1] | United Kingdom, 229 hospitals with consultant-led maternity units (UKOSS) | Feb 2005 – Aug 2006 | Prospective national surveillance, 57 women among 1,132,964 maternities | Swansea criteria plus clinical assessment | Incidence 5.0 per 100,000 maternities (95% CI 3.8–6.5); 55 cases (90%) confirmed by criteria plus clinical assessment and 2 (3%) by clinical assessment alone, giving 97% agreement (kappa 0.78); percentages refer to all notified cases (a denominator of 61 reproduces both the percentages and the kappa statistic); 60% intensive care, 15% specialist liver unit, 1 transplant; case fatality 1.8% (95% CI 0–9.4); perinatal mortality 104 per 1000 births (95% CI 43–203); twin pregnancy 18%; body mass index <20 in 20% |
| Goel et al., 2011 [22] | India, Vellore | 1998–2006 | Retrospective biopsy-anchored validation; 34 biopsied, 10 excluded, 24 analysed | Liver biopsy (19 postnatal, transjugular; 5 immediately post-mortem), within 11 days of delivery | Swansea criteria sensitivity 100%, specificity 57%, positive predictive value 85%, negative predictive value 100% for hepatic microvesicular steatosis. Two-page correspondence; 17 of 24 had microvesicular steatosis, all of whom met the criteria, as did 3 of the 7 without steatosis (Table S6); the figures have not been independently replicated; the investigators describe antenatal application of the thirteen non-histological items [21] |
| Nelson et al., 2013 [7] | United States, Parkland Hospital | 1975–2012 | Retrospective cohort, 51 women | Institutional clinical criteria | Persistent nausea and vomiting 57%, hypertension 57%, abdominal pain 53%, more than 90% with at least one; impaired procoagulant synthesis in more than three-quarters; 50% transfused; stillbirth 120 per 1000 pregnancies; 2 maternal deaths; |

| Study | Country / setting | Period | Design and size | Diagnostic basis | Principal quantitative findings |
|---|---|---|---|---|---|
| | | | | | clinical recovery in 3–4 days with most laboratory values normalising within 7–10 days |
| Nelson et al., 2014 [72] | United States, Parkland Hospital | — | Retrospective haemostatic study, 51 women | Institutional clinical criteria | 80% classified as having unequivocal disseminated intravascular coagulation at delivery (mean score 5.9 ± 1.8), with persisting dysfunction for 4–5 days postpartum |
| Lau et al., 2010 [44] | Taiwan, tertiary centre | 22 years | Retrospective review, 18 women | Clinical and laboratory criteria | 6 women (33%) diagnosed antepartum and 12 (67%) postpartum; 2 maternal deaths (11%); acute renal failure 83%, hypoglycaemia 61%, disseminated intravascular coagulation 61% |
| Vigil-De Gracia, 2001 [69] | Panama | Jan 1996 – Dec 1999 | Comparative retrospective series, 10 AFLP versus 75 HELLP syndrome | Clinical and laboratory criteria | AFLP characterised by hypoglycaemia, hypocholesterolaemia, hypotriglyceridaemia, raised serum transaminase activity and low antithrombin III; disseminated intravascular coagulation, renal insufficiency, ascites and encephalopathy more frequent than in HELLP syndrome; greater parity in HELLP syndrome ($p < 0.006$) |
| Cheng et al., 2014 [51] | China, Nanchang | 10 years | Retrospective series, 32 women | Clinical and laboratory criteria | Jaundice in all 32, nausea and vomiting 28, malaise 25, ascites 24; acute renal failure 26, hepatic encephalopathy 17, infection 10, postpartum haemorrhage 7 |
| Xiong et al., 2015 [78] | China, Beijing | — | Retrospective follow-up, 42 diagnosed and 25 followed | Clinical and laboratory criteria | 25 of 42 diagnosed patients followed; mean follow-up 54.5 months (range 6.5–181); ultrasound normal in 12, coarsened echo-pattern in 10, mild-to-moderate fatty infiltration in 3; no cirrhosis or nodules; liver and renal function normalised in all |
| Gao et al., 2018 [14] | China, four tertiary hospitals | Jan 2009 – Apr 2014 | Multicentre retrospective cohort, 133 women | Swansea criteria | Maternal mortality 16.5% (22/133); fetal mortality 22.4% (36 of 161 fetuses); postpartum diagnosis in 13 (9.8%), associated with worse maternal outcome ($p < 0.01$); abnormal glucose in 89 (66.9%): hypoglycaemia in 76 (57.1%) and hyperglycaemia in 13 (9.8%); independent predictors of maternal death: previous termination of pregnancy (OR 1.958, 95% CI 1.133–3.385), total bilirubin (OR 1.009, 95% CI 1.003–1.014), creatinine (OR 1.010, 95% CI 1.003–1.017) |
| Lamprecht et al., 2018 [73] | Australia, quaternary obstetric hospital | 15 years | Retrospective series, 17 women | Swansea criteria | Pre-existing gastrointestinal disease in 6 of 17 (35%), inflammatory bowel disease in 5; antithrombin III low, consistent with previous reports; no recurrence in 9 subsequent pregnancies to 7 women |
| Zhong et al., 2020 [34] | China, Changsha | 2009–2015 | Retrospective case–control; 41 AFLP versus 172 controls selected by diagnosis from 28,800 records | Swansea criteria as reference standard | Approximately 3 days before the final Swansea-based diagnosis (not necessarily first presentation): 0% met ≥6 criteria, 19.5% met 5, 75.6% met 4, 4.9% met 3; five-variable model (gastrointestinal symptoms with aminotransferase, bilirubin, bile acid and clotting-time abnormalities) sensitivity 97.6%, specificity 97.1%, positive and negative predictive values 88.9% and 99.4% (case–control sampling; cannot be directly transported to a lower-prevalence triage population without recalibration); prothrombin time >14 s in 40 of 41 (97.6%) versus 7 of 172 controls (4.1%); glucose |

| Study | Country / setting | Period | Design and size | Diagnostic basis | Principal quantitative findings |
|---|---|---|---|---|---|
| | | | | | <72 mg/dL in 5 of 41 (12.2%) versus 3 of 172 controls (reported as 2.3%; 1.7% by calculation); AFLP incidence 0.14% of records screened; maternal mortality lower and Apgar scores higher with model-prompted (n = 23) than with Swansea-based (n = 18) delivery, in a non-randomised comparison |
| Ang et al., 2021 [40] | Taiwan, single tertiary centre | Jun 2004 – Apr 2020 | Retrospective comparative cohort, 21 AFLP versus 80 HELLP syndrome | Swansea criteria (AFLP), Tennessee classification (HELLP) | See Table S2 for laboratory values; pre-eclampsia 23.8% versus 95.0%; jaundice 85.7% versus 13.8%; acute kidney injury 61.9% versus 15.0%; disseminated intravascular coagulation 66.7% versus 8.8%; sepsis 47.6% versus 10.0% |
| Meng et al., 2021 [38] | China, Shandong | — | Retrospective cohort, 106 women | Clinical and laboratory criteria | Anorexia 56.6%, vomiting 48.1%, nausea 46.2%, abdominal pain 30.2%, jaundice 23.6%, polydipsia or polyuria 9.4%, hepatic encephalopathy 7.5%; total bilirubin 134.1 ± 102.8 μmol/L with direct bilirubin 82.5 ± 60.9 μmol/L; urate 495.3 ± 157.5 μmol/L; creatinine 169.9 ± 95.7 μmol/L; fibrinogen 1.6 ± 1.3 g/L; MELD score predicted maternal mortality with AUC 0.948 (95% CI 0.904–0.992) |
| Tan et al., 2022 [13] | China, Beijing | Jan 2008 – Jan 2018 | Retrospective cohort, 73 women among 24,054 obstetric and intensive care admissions (0.303%) | Clinical diagnosis; Swansea criteria applied by retrospective rescoring | 66 women (90.4%) met ≥6 criteria and 7 (9.6%) did not; maternal fatality 4.1% (3/73), fetal 17.3% (14/81); Swansea score predicted hepatic encephalopathy (AUC 0.773, cut-off 8), acute renal failure (AUC 0.796, cut-off 8), continuous renal replacement therapy (AUC 0.805, cut-off 8), stillbirth (AUC 0.894, cut-off 10); across eleven complications and treatment requirements, AUCs ranged from 0.694 to 0.894 and in-sample optimal cut-offs from 7 to 10 |
| Li et al., 2023 [6] | China, two tertiary centres | Jan 2012 – May 2022 | Retrospective cohort, 298 diagnosed and 290 analysed | Swansea criteria | Plasma exchange in 79 (27.2%); maternal mortality 50/290 (17.2%); fetal death 78 (26.9%); fetal distress 130 (44.8%); hepatic encephalopathy 81 (27.9%); infection 60 (20.7%); continuous renal replacement therapy 107 (36.9%); intervals from symptom onset to hospitalisation and to delivery were associated with death |
| Peng et al., 2024 [74] | China, two centres | — | Retrospective prognostic cohort, 78 women | Clinical, laboratory and Swansea criteria | Three-month mortality 14.1% (11/78); five-variable index (total bilirubin, INR, creatinine, platelets, artificial liver support) measured 72 h postpartum: AUC 0.874 versus MELD 0.781, MELD-Na 0.774, iMELD 0.744 |
| Lan et al., 2025 [5] | China, Guangzhou | Jan 2010 – Oct 2022 | Retrospective cohort, 133 women | Swansea criteria | Acute liver failure in 33 (24.8%); maternal mortality 5/133 (3.8%); no liver biopsies performed; ultrasonographic fatty change in 20 (15.0%) with 4 more showing slight parenchymal enhancement on computed tomography; total bilirubin predicted acute liver failure (AUC 0.80, 95% CI 0.72–0.88, cut-off 61.58 μmol/L), prothrombin time (AUC 0.77, 95% CI 0.68–0.86, cut-off 16.70 s) |
| Zhai et al., 2026 [4] | China, Guangzhou | Jul 2017 – Jul 2023 | Retrospective cohort, 92 women | Modified Swansea criteria | Intensive care admission 61.95%; multiorgan dysfunction 23.9%; postpartum haemorrhage 15.2%; disseminated intravascular coagulation 6.5%; |

| Study | Country / setting | Period | Design and size | Diagnostic basis | Principal quantitative findings |
|---|---|---|---|---|---|
| | | | | | hepatic encephalopathy 4.3%; pancreatitis 3.3%; maternal death 1.09% (1/92); areas under the curve for a composite adverse outcome (intensive care admission or critical maternal complications, including coagulopathy and disseminated intravascular coagulation): PT and INR each 0.837 (95% CI 0.751–0.924; p < 0.001), fibrinogen 0.320 (95% CI 0.208–0.432; p = 0.004; lower values associated with the outcome) and antithrombin III 0.420 (95% CI 0.294–0.546; p = 0.202) |
| Ahmed et al., 2026 [50] | Pakistan, Karachi | Nov 2024 – Apr 2025 | Retrospective observational series, 73 women | Swansea criteria | Nausea and vomiting 87.7%, malaise 76.7%, jaundice 64.4%, coagulopathy 42.5%, acute kidney injury 23.3%; stillbirth 17.8%, early neonatal death 6.8%, perinatal mortality 24.6% |
| Bourque and Mahone, 2025 [33] | Systematic literature review | — | 14 women with second-trimester AFLP or compatible biopsy | Swansea criteria or biopsy | Diagnosis between 20 and 27 weeks and 6 days; 13 of 14 met the Swansea criteria; 10 of 11 biopsied had microvesicular steatosis; 50% primigravid; 1 maternal, 7 fetal and 3 neonatal deaths |
| Suzuki et al., 2025 [42] | Japan, pooled review of published case reports | — | 102 patients from 93 articles | Reported clinical diagnosis | Antithrombin 23.0% (IQR 17.0–33.0); fibrinogen 82.0 mg/dL (IQR 52.5–153.5); PT-INR 1.59 (IQR 1.31–2.02); APTT 47.5 s (IQR 28.2–97.5); platelets 16.1 × $10^4$/μL (IQR 11.1–19.2), with approximately 70% at or above 12 × $10^4$/μL; thrombin–antithrombin complex 60.0 ng/mL; fibrinogen/fibrin degradation products 49.2 μg/mL |
| Varotsis et al., 2026 [48] | Systematic review | — | 27 studies, 77 pregnancies with genetic or biochemical testing | Reported clinical diagnosis | Confirmed fetal fatty acid oxidation disorder in 27 (35.1%): LCHAD deficiency 22 (81.5%), MCAD deficiency 3 (11.1%), SCAD deficiency 1 (3.7%), CPT-I deficiency 1 (3.7%); gastrointestinal manifestations in 87.8% |

AFLP, acute fatty liver of pregnancy; APTT, activated partial thromboplastin time; AUC, area under the receiver operating characteristic curve; CI, confidence interval; CPT-I, carnitine palmitoyltransferase I; HELLP, haemolysis, elevated liver enzymes and low platelets; INR, international normalised ratio; IQR, interquartile range; LCHAD, long-chain 3-hydroxyacyl-coenzyme A dehydrogenase; MCAD, medium-chain acyl-coenzyme A dehydrogenase; MELD, Model for End-stage Liver Disease; MELD-Na, MELD incorporating serum sodium; iMELD, integrated MELD; OR, odds ratio; PT, prothrombin time; SCAD, short-chain acyl-coenzyme A dehydrogenase; UKOSS, United Kingdom Obstetric Surveillance System.

**Table S2.** Laboratory values in acute fatty liver of pregnancy organised by functional domain, with a single-centre comparison against HELLP syndrome

| Analyte | AFLP versus HELLP syndrome: single-centre comparison, 21 versus 80 women [40] [a] | AFLP: other cohorts and pooled case reports [b] |
|---|---|---|
| **Hepatic synthetic function and coagulation** | | |
| Prothrombin time (s) | 14.1 versus 9.5; $p < 0.001$ | Mean 16.7 ± 7.5 (range 8.5–80.0), n = 133 [5]; mean 17.1 ± 8.4, abnormal in 73.9%, n = 92 [4] |
| Activated partial thromboplastin time (s) | 38.5 versus 27.9; $p < 0.001$ | Mean 45.6 (range 18.4–138.5), n = 133 [5] [c]; median 47.5 (IQR 28.2–97.5), pooled case-report series of 102 patients [42] |

| Analyte | AFLP versus HELLP syndrome: single-centre comparison, 21 versus 80 women [40] [a] | AFLP: other cohorts and pooled case reports [b] |
|---|---|---|
| INR | — | Median 1.59 (IQR 1.31–2.02), pooled case-report series of 102 patients [42]; mean 1.6 ± 0.8, abnormal in 73.6%, n = 92 [4] |
| Fibrinogen | 193 versus 408.1 mg/dL; $p < 0.001$ | Median 82.0 mg/dL (IQR 52.5–153.5), pooled case-report series of 102 patients [42]; mean 1.7 ± 1.0 g/L (range 0.2–6.8), n = 133 [5]; mean 1.6 ± 1.3 g/L, n = 106 [38] |
| Antithrombin activity | Not reported | Median 23.0% (IQR 17.0–33.0), pooled case-report series of 102 patients [42] |
| Albumin (g/L) | Not reported | Mean 28.2 ± 5.7, n = 106 [38] |
| **Hepatic excretory function** | | |
| Total bilirubin | 4.4 versus 0.9 mg/dL; $p < 0.001$ | Mean 99.1 μmol/L (range 4.7–401.5), n = 133 [5] [c]; mean 134.1 ± 102.8 μmol/L, n = 106 [38] |
| Direct bilirubin | — | Mean 84.3 μmol/L (range 4.1–288.1), n = 133 [5] [c]; mean 82.5 ± 60.9 μmol/L, n = 106 [38] |
| **Hepatocellular injury** | | |
| Aspartate aminotransferase | 257 versus 124 IU/L; $p = 0.003$ | Mean 254.0 ± 245.9 U/L (range 5.6–1832.3), n = 133 [5] |
| Alanine aminotransferase | 316 versus 135 IU/L; $p = 0.006$ | Mean 287.0 ± 280.0 U/L (range 14.0–1568.1), n = 133 [5]; mean 292.2 ± 281.6 U/L, n = 106 [38] |
| Lactate dehydrogenase | Not significantly different | — |
| **Hepatic metabolic function** | | |
| Glucose | 85.0 versus 98.5 mg/dL; $p < 0.001$ | Mean 4.4 ± 2.0 mmol/L, n = 106 [38] |
| Ammonia | Not significantly different | — |
| **Renal function and urate** | | |
| Creatinine | 1.8 versus 0.8 mg/dL; $p < 0.001$ | Mean 169.9 ± 95.7 μmol/L, n = 106 [38] |
| Uric acid | Not significantly different | Mean 495.3 ± 157.5 μmol/L, n = 106 [38] |
| **Haematological indices** | | |
| Platelets | 174 versus 89 × $10^3$/μL; $p < 0.001$ | Median 16.1 × $10^4$/μL (IQR 11.1–19.2), pooled case-report series of 102 patients [42]; mean 150.3 × $10^9$/L, n = 106 [38] [c] |
| Leucocytes | — | Mean 15.1 ± 5.6 × $10^9$/L, n = 106 [38]; mean 15.0 ± 8.8 × $10^9$/L (range 5.1–86.6), n = 133 [5] |
| **Placental angiogenic markers** | | |
| Soluble fms-like tyrosine kinase-1 | — | Median 57,570 pg/mL (range 31,609–147,170), n = 6, versus 9713 pg/mL in HELLP syndrome, n = 48 [36]; median 77,762 pg/mL (IQR 45,044–116,657), n = 12 antepartum cases [66] |
| Placental growth factor | — | Free PlGF median 197 pg/mL (range 127–487), n = 6 [36]; total PlGF median 2054 pg/mL, n = 12 [66] |

[a] Data from Ang et al. [40] (source Table 3; published under a CC BY 4.0 licence), re-organised by the present authors; fibrinogen and ammonia were reported as mean ± standard deviation and the remaining analytes as median (interquartile range), with $p$ values as reported. [b] Mean ± standard deviation, median (interquartile range) or range, as reported by the original authors. [c] Dispersion values printed in the source are omitted because they are implausible if interpreted as standard deviations: ± 7.4 μmol/L for total bilirubin and ± 5.9 μmol/L for direct bilirubin are incompatible with the reported ranges, ± 45.6 s for APTT is identical to the mean, and ± 7.4 × $10^9$/L for platelets is

implausibly small; means are shown as printed. —, not extracted. Direct comparison across rows is limited by differences in assay, units and timing of sampling. Conversion factors: bilirubin, 1 mg/dL = 17.1 μmol/L; creatinine, 1 mg/dL = 88.4 μmol/L; glucose, 1 mmol/L = 18.0 mg/dL; fibrinogen, 1 g/L = 100 mg/dL; platelets, $1 \times 10^3/\mu L = 1 \times 10^9/L$; aminotransferase activities in IU/L and U/L are equivalent. AFLP, acute fatty liver of pregnancy; APTT, activated partial thromboplastin time; HELLP, haemolysis, elevated liver enzymes and low platelets; INR, international normalised ratio; IQR, interquartile range; PlGF, placental growth factor.

**Table S3.** Conditions in the second half of pregnancy that can fulfil Swansea-type features, and the investigations that discriminate them

| Condition | Features shared with acute fatty liver of pregnancy | Discriminating features and investigations | Supporting evidence |
|---|---|---|---|
| HELLP syndrome and pre-eclampsia with severe features | Vomiting, abdominal pain, raised transaminases, raised bilirubin, leucocytosis, raised urate, renal impairment, coagulopathy | Marked thrombocytopenia with comparatively preserved synthetic function; hypertension and proteinuria in the great majority; fibrinogen typically elevated rather than reduced; hypoglycaemia and jaundice uncommon | Comparative cohort with full laboratory panel [40]; approximately half of women with AFLP are not thrombocytopenic at presentation [43]; direct evaluation showing that HELLP syndrome also meets the Swansea criteria [25]; in a multicentre US acute liver failure registry that included no cases of hepatitis A–E, the criteria were satisfied by every woman with pregnancy-associated liver disease and by virtually all women with other causes [32] |
| Hepatitis E virus infection | Jaundice, vomiting, coagulopathy, encephalopathy, renal impairment, leucocytosis | Anti-HEV IgM and HEV RNA; regional epidemiology; hypoglycaemia less characteristic; markedly higher transaminases | Pooled maternal case-fatality risk 20.8% (95% CI 16.6–25.3) across 47 studies and 3968 women, rising to 61.2% with fulminant hepatic failure [26]; fulminant hepatic failure RR 2.7 (95% CI 1.7–4.2) and maternal death RR 6.0 (95% CI 2.7–13.3) versus non-HEV hepatitis [27]; direct evidence that viral acute liver failure fulfils the Swansea criteria [25] |
| Hepatitis A and B virus infection | Jaundice, transaminitis, coagulopathy | Serology (anti-HAV IgM, HBsAg, anti-HBc IgM); in a comparative series, hypertension and ascites favoured pregnancy-associated liver disease over viral hepatitis | Comparative study of 87 jaundiced pregnant women with pregnancy-associated liver disease or acute viral hepatitis, two-thirds of the latter due to hepatitis E [76] |
| Herpes simplex virus hepatitis | Vomiting, abdominal pain, coagulopathy, encephalopathy, marked transaminitis | Fever, often with anicteric hepatitis; mucocutaneous lesions may be absent; HSV PCR; empirical aciclovir | Fever was the initial symptom in all 24 reported pregnancies, with 6 maternal deaths [28] |
| Thrombotic thrombocytopenic purpura | Thrombocytopenia, haemolysis, renal impairment, neurological features | ADAMTS13 activity below 10%; prominent neurological involvement with comparatively less severe renal injury; PLASMIC score ≥6; hepatic synthetic function preserved | Contemporary review of pregnancy-associated thrombotic thrombocytopenic purpura, which explicitly contrasts it with AFLP [29] |

| Condition | Features shared with acute fatty liver of pregnancy | Discriminating features and investigations | Supporting evidence |
| --- | --- | --- | --- |
| Complement-mediated thrombotic microangiopathy | Thrombocytopenia, haemolysis, severe acute kidney injury | Typically postpartum onset; ADAMTS13 preserved or only mildly reduced; severe renal injury predominates; complement evaluation | Same source [29] |
| Haemophagocytic lymphohistiocytosis | Fever, cytopenias, transaminitis, hyperbilirubinaemia, coagulopathy, hypofibrinogenaemia | Ferritin above 500 ng/mL in 92% of reported cases, hypertriglyceridaemia in 49%, hypofibrinogenaemia in 19%; haemophagocytosis; progressive rather than post-delivery resolution; HLH-2004 criteria | Review of 81 pregnancies with maternal mortality 22% and fetal mortality 40% [30]; case report of haemophagocytic lymphohistiocytosis presenting as AFLP [31] |
| Drug-induced liver injury, particularly paracetamol | Vomiting, transaminitis, coagulopathy, encephalopathy, renal impairment | Exposure history; very high transaminases with a characteristic time course; paracetamol concentration; response to acetylcysteine | Paracetamol accounted for 21 of 35 non-pregnancy-specific cases of acute liver injury or failure in pregnancy in a multicentre US registry [32] |
| Intrahepatic cholestasis of pregnancy | Raised transaminases, raised bilirubin, pruritus | Markedly raised total bile acids with preserved synthetic function and no hypoglycaemia or encephalopathy | Included as a control condition in the only pre-threshold diagnostic study identified, in which bile acids formed part of the discriminating model [34] |
| Sepsis-associated cholestasis and ischaemic hepatitis | Jaundice, transaminitis, coagulopathy, renal impairment | Documented infection or haemodynamic insult; blood cultures; lactate; pattern of transaminase rise and fall | No pregnancy-specific quantitative evidence was identified; this entry rests on general hepatological principle and should be regarded as a knowledge gap |

Reference numbers in this table refer to the main manuscript reference list. ADAMTS13, a disintegrin and metalloproteinase with a thrombospondin type 1 motif, member 13; AFLP, acute fatty liver of pregnancy; CI, confidence interval; HAV, hepatitis A virus; HBc, hepatitis B core antigen; HBsAg, hepatitis B surface antigen; HELLP, haemolysis, elevated liver enzymes and low platelets; HEV, hepatitis E virus; HLH, haemophagocytic lymphohistiocytosis; HSV, herpes simplex virus; IgM, immunoglobulin M; PCR, polymerase chain reaction; RR, relative risk.

**Table S4.** Evidence linking fetal fatty acid oxidation defects to maternal liver disease in pregnancy

| Study | Design | Population | Principal finding | Interpretation |
| --- | --- | --- | --- | --- |
| Wilcken et al., 1993 [54] | Case series | Eleven pregnancies in five mothers; six infants with LCHAD deficiency | Every pregnancy carrying an LCHAD-deficient infant was complicated by features such as fatty liver or HELLP syndrome, whereas pregnancies with unaffected infants were largely uncomplicated | Early evidence that maternal liver disease is associated with an affected fetal genotype rather than with maternal carrier status alone |
| Treem et al., 1994 [55] | Index case report | One mother–infant pair | Woman with AFLP whose apparently normal full-term infant presented at 4 months with hypoglycaemia, coma and profound hepatic steatosis and proved to have LCHAD deficiency; the mother was a heterozygous carrier | Most detailed early index case; the original authors proposed the defect as explaining "some cases" of AFLP |
| Sims et al., 1995 [56] | Molecular characterisation | Three families | Two children homozygous for G1528C, a G→C transversion substituting glutamine for glutamic acid at residue 474 of the mature $\alpha$-subunit; one compound heterozygote with a | Early delineation of the molecular basis of isolated LCHAD deficiency; the designation E474Q was applied to this substitution in later reports and is retained |

| Study | Design | Population | Principal finding | Interpretation |
| --- | --- | --- | --- | --- |
| | | | premature termination codon (C1132T) | when those studies are described; in current nomenclature c.1528G>C (p.Glu510Gln) in *HADHA*, the gene encoding the MTP $\alpha$-subunit (NM_000182.5), numbered on the precursor protein |
| Ibdah et al., 1999 [57] | Case series with molecular genotyping | 24 children with deficient LCHAD activity: 19 with isolated LCHAD deficiency and 5 with complete MTP deficiency | 79% of heterozygous mothers carrying a fetus with the E474Q variant developed AFLP or HELLP syndrome; of the 19 children with isolated LCHAD deficiency, 8 were homozygous and 11 compound heterozygous for E474Q; the 5 children with complete MTP deficiency carried no E474Q allele, and none of their mothers had liver disease | The strongest evidence for the association, with an internal genotype–phenotype dissociation that limits generalisation |
| den Boer et al., 2000 [60] | Population screening | 2047 Guthrie cards and 113 women with previous HELLP syndrome | Carrier frequency of the c.1528G>C variant approximately 1 in 680 in the Dutch population; one carrier among 113 women with HELLP syndrome | Heterozygous carriage is not a major contributor to HELLP syndrome |
| Yang et al., 2002 [58] | Cohort of consecutive referred samples, 1997–2001 | 108 samples; 27 pregnancies with AFLP and 81 with HELLP syndrome | Variants causing LCHAD deficiency in 5 families (19%) among the AFLP pregnancies; only one woman with HELLP syndrome was heterozygous and no variant was detected in her newborn | Independent confirmation of the association and of its restriction to AFLP rather than HELLP syndrome; the published 95% confidence interval (9–54%) is wider than the exact binomial interval for 5 of 27 (6–38%) |
| Browning et al., 2006 [59] | Matched case–control | 50 infants with fatty acid oxidation defects and 1250 matched controls | Maternal liver disease in 16.00% of affected pregnancies versus 0.88% in the general population (odds ratio 20.4, 95% CI 7.82–53.2), and an 18.1-fold increase against the matched controls; approximately 50-fold excess for long-chain and 12-fold for short- and medium-chain defects | Extends the association across the whole spectrum of fatty acid oxidation defects; also shows that 84% of affected pregnancies produce no maternal liver disease |
| Mütze et al., 2007 [61] | Genotyping study | 103 women with HELLP syndrome, 82 children, 21 fathers, 103 controls | The E474Q variant was not detected | Neither maternal nor fetal E474Q is a relevant factor in HELLP syndrome |
| Natarajan and Ibdah, 2018 [62] | Mechanistic review | — | Proposes that 3-hydroxy fatty acid intermediates accumulating in the placenta reach the maternal circulation and impair hepatocyte mitochondrial function | Provides the mechanistic bridge between the genetic association and the hepatic lesion. It presents no new data; the placental and mitochondrial limbs rest on human placental mitochondrial studies and murine MTP knockouts, while the final hepatocellular step remains hypothetical |
| Varotsis et al., 2026 [48] | Systematic review | 27 studies, 77 pregnancies with | Confirmed fetal defect in 27 (35.1%): LCHAD deficiency 22 (81.5%), MCAD | Best current pooled estimate, derived from |

| Study | Design | Population | Principal finding | Interpretation |
|---|---|---|---|---|
| | | genetic or biochemical testing | deficiency 3 (11.1%), SCAD deficiency 1 (3.7%), CPT-I deficiency 1 (3.7%); stillbirth or infant death in 14 of 22 LCHAD-deficient cases (63.6%; 4 stillbirths and 10 infant deaths) | selectively tested pregnancies rather than a population prevalence; approximately two-thirds of tested AFLP pregnancies have no demonstrable fetal defect |

Reference numbers correspond to the main manuscript reference list. AFLP, acute fatty liver of pregnancy; CI, confidence interval; CPT-I, carnitine palmitoyltransferase I; HELLP, haemolysis, elevated liver enzymes and low platelets; LCHAD, long-chain 3-hydroxyacyl-coenzyme A dehydrogenase; MCAD, medium-chain acyl-coenzyme A dehydrogenase; MTP, mitochondrial trifunctional protein; SCAD, short-chain acyl-coenzyme A dehydrogenase.

**Table S5.** SANRA items and where each is addressed in the present narrative review (descriptive checklist)

The Scale for the Assessment of Narrative Review Articles (SANRA) [18] comprises six items. This table lists each item and indicates how and where it is addressed in the manuscript. It is provided as a descriptive checklist for editors and reviewers; no scores are assigned, and it is not a measure of quality or scientific validity.

| Item | Criterion | How the manuscript addresses it | Manuscript location |
|---|---|---|---|
| 1 | Justification of the article's importance for the readership | The clinical problem is quantified with incidence, intensive-care and mortality figures from national surveillance and contemporary cohorts, and the specific gap—behaviour of the diagnostic criteria across the course of the illness—is stated | Section 1, paragraphs 1–4 |
| 2 | Statement of concrete aims or formulation of questions | Three explicit objectives are stated: to appraise the operating characteristics of the Swansea criteria as a function of disease stage, to appraise candidate early markers, and to specify design principles and validation requirements for early-disease criteria | Section 1, paragraph 5 |
| 3 | Description of the literature search | PubMed/MEDLINE is specified as the bibliographic database searched, Europe PMC as an additional search and full-text access interface, and Crossref and publisher records as supplementary resources for locating sources and verifying bibliographic details; the five conceptual domains and their relation to the review's objectives, backward and forward citation tracking, the relevance-based selection of sources, the language and publication span of the sources cited and the preference for primary studies for quantitative claims are described; the narrative, non-exhaustive nature of the process is stated explicitly, and its limited reproducibility is acknowledged (Section 8.2); the date of the last search, the complete search strings, any limits applied and record counts are not reported, and Embase, Scopus and the Cochrane Library were not searched separately | Section 1.1 |
| 4 | Referencing | Every substantive claim carries a reference, and primary studies are cited in preference to reviews for specific quantitative findings. All references were verified against their original bibliographic records, and quantitative statements against the primary sources; where a printed figure appears inconsistent or its unit could not be resolved, this is stated (Sections 4.2 and 6.4; Tables S1, S2 and S4) | Throughout; Reference list |
| 5 | Scientific reasoning | Evidence is weighted by design and sample size, and contradictory evidence is sought and reported rather than omitted: the prognostic antithrombin area under the curve of 0.420, the 90.4% criterion fulfilment on retrospective rescoring, the presence of hypoglycaemia in only 12.2% of women approximately three days | Sections 1.1, 3–6, 8; Tables 2 and 3 |

| Item | Criterion | How the manuscript addresses it | Manuscript location |
|---|---|---|---|
| | | before the final diagnosis, and the absence, to our knowledge, of experimental evidence that earlier diagnosis improves survival. Certainty is summarised qualitatively in Tables 2 and 3, using criteria defined in Section 1.1 | |
| 6 | Appropriate presentation of relevant endpoint data | Sensitivities, specificities, predictive values, odds and relative risks with confidence intervals, absolute event rates, areas under the curve and laboratory values are reported throughout, with an illustrative prevalence-dependence calculation | Sections 3–6; Tables 1–3; Tables S1, S2, S6 |

**Table S6.** Illustrative model-based calculations of predictive values across a range of disease prevalences

These are illustrative Bayes calculations, not empirical estimates or observed predictive values in any contemporary clinical population. Predictive values were calculated from the published sensitivity and specificity of each rule using Bayes' theorem, under the unverified assumption that sensitivity and specificity remain unchanged across populations, which is itself questionable given the differences in spectrum between the derivation samples and a triage population. Published predictive values cannot be directly transported to a lower-prevalence triage population without recalibration. The Swansea figures use the sensitivity of 100% and specificity of 57% reported against biopsy-proven microvesicular steatosis [22]; the second column pair uses the sensitivity of 97.6% and specificity of 97.1% reported for the combination of gastrointestinal symptoms with aminotransferase, bilirubin, bile acid and clotting-time abnormalities, measured approximately three days before the final Swansea-based diagnosis in a case–control sample with the Swansea criteria as reference standard [34].

| Prevalence of AFLP in the population assessed | Swansea criteria: PPV | Swansea criteria: NPV | Pre-threshold model: PPV | Pre-threshold model: NPV |
|---|---|---|---|---|
| 1% | 2.3% | 100% | 25.4% | 99.98% |
| 3.5% (assumed; women with abnormal liver tests in pregnancy in one prospective cohort [3]) | 7.8% | 100% | 55.0% | 99.91% |
| 10% | 20.5% | 100% | 78.9% | 99.73% |
| 25% | 43.7% | 100% | 91.8% | 99.18% |
| 50% | 69.9% | 100% | 97.1% | 97.59% |
| 71% (prevalence in the biopsy validation sample [22]) | 85.1% | 100% | 98.8% | 94.29% |

The 85.1% value in the final row reproduces the positive predictive value of 85% reported in the original validation, in which the counts were 17 true positives, 0 false negatives, 3 false positives and 4 true negatives (prevalence 70.8%). The 100% negative predictive value of the Swansea criteria at every prevalence follows from the reported sensitivity of 100%, not from the high prevalence of the validation sample, and the small validation sample does not establish that a score below six excludes early disease. AFLP, acute fatty liver of pregnancy; NPV, negative predictive value; PPV, positive predictive value.
